# Population pharmacokinetic model for meropenem in pediatric patients: a systematic review

**DOI:** 10.3389/fmicb.2026.1897237

**Published:** 2026-09-17

**Authors:** Huaping Gao, Qing Tang, Leying Wu, Yuhua Zhao, Zhenwei Yu, Hongbo Xia

**Affiliations:** 1 Department of Pharmacy, Sir Run Run Shaw Hospital, School of Medicine, Zhejiang University, Hangzhou, China; 2 Research Center for Clinical Pharmacy, Zhejiang University, Hangzhou, China; 3 Affiliated Xiaoshan Hospital, Hangzhou Normal University, Hangzhou, China

**Keywords:** drug clearance, meropenem, neonate, pediatric, pharmacokinetic, preterm, therapeutic drug monitoring, volume of distribution

## Abstract

**Background:**

The pharmacokinetics of meropenem in pediatric patients have large interindividual variability. Model-informed precision dosing (MIPD) is a promising way to ensure optimal exposure but relies on a population pharmacokinetic (PPK) model. This review systemically synthesized the available PPK model for meropenem in pediatric patients to facilitate MIPD in these patients.

**Methods:**

Four electronic databases were searched for eligible studies that reported the use of the meropenem PPK model for pediatric patients. The study design information, patient characteristic demography, PPK modeling strategy and final PPK parameter estimates were extracted and reviewed.

**Results:**

Twenty studies published from 2006 to 2025 were included in the analysis. Fourteen studies had prospective designs, and four studies were international multicenter studies. Seven studies applied an intensive sampling strategy. Pediatric patients with various characteristics were included, and most had severe infections or were admitted to the ICU. Four studies focused on patients with organ support, mainly renal replacement and extracorporeal membrane oxygenation. The sample sizes were usually small (sample sizes no more than 50). Fourteen studies ultimately obtained the 2-CMT model, and the remaining studies used the 1-CMT model, except for one 3-CMT model. Body weight, age and renal function indicators were the most common covariates. The typical CL values ranged from 0.50–13.22 L/h or 0.122–0.526 L/h/kg. The Vcs for the 2-CMT models ranged from 0.969–21.4 L or 0.272–0.57 L/kg. Most models underwent internal validation, but only one study performed external validation.

**Conclusion:**

This study successfully integrated the current meropenem PPK models for pediatric patients, which would benefit the clinical application of MIPD. Clinicians should select appropriate models on the basis of patient characteristics carefully, and therapeutic drug monitoring is important at the current stage. External validation and well-designed studies on specific subpopulations, such as patients with organ support, neonates, and specific disease status, are needed.

**Systematic review registration:**

PROSPERO, identifier (CRD420261481301).

## Introduction

Meropenem is recognized as the first-line treatment for severe infection (Tamma et al., 2022; Mu et al., 2025). It has good *in vitro* activity against a broad spectrum of pathogens, including ESBL-producing *Enterobacterales*, *Pseudomonas aeruginosa* and *Acinetobacter baumannii* and *methicillin-resistant Staphylococcus aureus* (Guo et al., 2022). Although resistance to meropenem has increased in recent decades, it remains the cornerstone of anti-infection therapy (Meschiari et al., 2024; Perez et al., 2025). In many trials evaluating the efficacy of novel antibiotics for drug-resistant pathogens, meropenem has been selected for comparison (Carmeli et al., 2025; Martin-Loeches et al., 2022). In terms of the safety profile, meropenem has a greater tolerance dose range and less neurotoxicity than imipenem does (Tan et al., 2021; Salmon-Rousseau et al., 2020). Thus, it is welcomed for pediatric patients with severe infection. It has been reported that the consumption of meropenem in pediatric patients is high (Balkhy et al., 2019).

In the era of drug resistance, antibiotics should be dosed under the guidance of the pharmacokinetic/pharmacodynamic (PK/PD) theory (Alikhani et al., 2025; Rao and Landersdorfer, 2021). The PK/PD index that is associated with the efficacy of meropenem is the time fraction of free drug above the minimum inhibitory concentration during a dosing interval (*f*T% > MIC). The *f*T% > MIC of meropenem should be greater than 40%–70% for bacteriostatic effects, and an *f*T% > MIC of 100% is required for maximal effects in critically ill patients (Roberts et al., 2014). The target also depends on the infected microorganism and infection type. Pediatric patients with severe infection always have unique pathophysiological conditions and high interindividual variability in PK (Goulooze et al., 2023). For example, sepsis was reported to be associated with subtherapeutic beta-lactam antibiotic concentrations in both adults and children and was often associated with augmented renal clearance (ARC), which is potentially related to increased cardiac output and renal blood flow (Bradley et al., 2025). Thus, the label dosing regimen of meropenem may often result in suboptimal exposure in children.

Model-informed precision dosing (MIPD) is a promising way to achieve optimal exposure for pediatric patients (Tang Girdwood et al., 2025). However, this approach highly relies on the robustness of the population pharmacokinetic (PPK) model. There are many published meropenem PPK models for pediatric patients, some of which have focused on special subpopulations. However, many models are limited in some aspects, and choosing an appropriate model when model-informed dosing is applied is difficult (Uster et al., 2021). Thus, we performed this systematic review to comprehensively compare the model characteristics, which would be helpful for future MIPD applications and research.

## Methods

This systematic review is conducted in accordance with the PRIMSA statement and is registered in PROSPERO (CRD420261481301).

### Literature search

Four electronic databases (PubMed, Web of Science, Embase, and the Cochrane Central Register of Controlled Trials) were searched to identify potentially eligible studies. The detailed search strategies are shown in Supplementary Table S1. The search time range was from the setup of the database to 2025. The language of publication was restricted to English. For the retrieved records, the reviewers performed initial assessments on the basis of the title and abstract, and irrelevant records were excluded. The full text was subsequently retrieved for eligibility. Two reviewers independently performed the literature search and then cross-checked the data. Any discrepancies were resolved with a third reviewer.

### Study inclusion

The titles/abstracts of the retrieved studies were screened, and the full texts of the remaining studies were assessed for eligibility. The inclusion criteria were as follows: (1) the study population was pediatric patients (aged under 18 years); (2) meropenem was administered intravenously; and (3) the meropenem PPK model was developed in the study. If the model focused on pediatric patients but included adult patients, it was also recognized as eligible.

However, the following exclusion criteria were also applied: (1) non-PPK studies, for example, meropenem pharmacokinetic parameters were calculated by noncompartmental analysis (NCA) rather than the PPK method; (2) a review or systematic review of previous meropenem PPK models; (3) a study focused on PPK model application; (4) a Physiologically Based Pharmacokinetic (PBPK) modeling study; (5) an external validation study of previous meropenem PPK models; (6) the study drug was not meropenem, such as meropenem-vaborbactam; and (7) key parameters of the PPK model were not reported.

#### Literature quality assessment

The quality of each included study was assessed according to the combined checklist based on the ClinPK statement and population pharmacokinetic–pharmacodynamic guidelines (Jamsen et al., 2014; Kanji et al., 2015). The checklist consisted of 35 criteria categorized into five domains: title/abstract, background, methods, results, and discussion/conclusion. The quality of the included studies was expressed as the compliance rate of the criteria. Two reviewers independently assessed the quality, and any discrepancies were resolved by a third reviewer.

### Data collection

Two reviewers independently performed data collection on the basis of electronic data item forms, and any discrepancies were resolved by a third reviewer. The following data were extracted: (1) study design information (first author and publication year, study design, country of study performed, sampling strategy, and meropenem assay method); (2) patient characteristic demography of the study enrolled/include patient group, number of included patients, meropenem plasma concentration points, age, sex, and weight distribution; (3) PPK modeling strategy (modeling software, covariate model method, and screened and identified covariates); (4) PPK parameter estimates (typical value of each parameter, covariate model, interindividual variability, residual error, and validation methods); and (5) the PK/PD target used for simulation and dosage regimen recommendations. The missing data were left empty, and the authors were not contacted.

### Covariate effect analysis

The effects of various variables on clearance were analyzed. The clearance parameters were mainly the clearance rate (CL) of the central department. For continuous covariates, the median value of the original study was used as the median, and the range of values across studies was used as the endpoint. To facilitate comparisons across different studies, the covariate effects reported in each model were uniformly converted into relative change rates of parameter estimates compared to the reference group. Then, forest plots were generated to visualize the effects of the identified covariates. Changes within the 80%–125% range were considered clinically insignificant.

## Results

### Literature search, study inclusion and quality assessment

The detailed literature search and study inclusion criteria are shown in Supplementary Figure S1. A total of 493 records were retrieved, and 20 studies reporting the meropenem PPK model for pediatric patients were ultimately eligible for analysis and included (Du et al., 2006; Bradley et al., 2008; Wang et al., 2020; Saito et al., 2021; Wang Y. et al., 2021; Zylbersztajn et al., 2021; Yonwises et al., 2021; Wu et al., 2022; Pokorná et al., 2024; Butragueño-Laiseca et al., 2024; Junior et al., 2024; Le et al., 2025; Van Den Anker et al., 2009; Ikawa et al., 2010; Ohata et al., 2011; Smith et al., 2011; Kongthavonsakul et al., 2016; Pettit et al., 2016; Cies et al., 2017; Ohata et al., 2019). The quality of the included studies was assessed by a 35-item checklist, and the detailed results are shown in Supplementary Table S2. Most had a high reporting item compliance rate (>85%), which indicated good quality.

### Design characteristics of the included studies

The design characteristics of the 20 included models are shown in Table 1. The studies are published from 2006 to 2025. Fourteen studies had prospective designs, and the remaining 6 studies were retrospective. In terms of the study region, the US was the most common, with 8 studies. Four studies were international multicenter studies. Seven studies applied an intensive sampling strategy. The detailed drug administration and blood collection information are shown in Supplementary Table S3. Fourteen studies used HPLC for the determination of meropenem, and the other four used LC–MS/MS methods.

**Table 1:** Study design characteristics of the available Meropenem population pharmacokinetic model for pediatric patients.

ID	Author	Study type	Publication year	Country	Sampling strategy	Assay
1	Du et al. (2006)	Retrospective	2006	USA	Sparse sampling	HPLC
2	Bradley et al. (2008)	Prospective	2008	USA	Sparse sampling	HPLC-UV
3	Van Den Anker et al. (2009)	Prospective	2009	Netherlands, Czech	Intensive sampling	HPLC-UV
4	Ikawa et al. (2010)	Retrospective	2010	Japan	NR	HPLC-UV
5	Ohata et al. (2011)	Prospective	2011	Japan	Sparse sampling	HPLC-UV
6	Smith et al. (2011)	Prospective	2011	USA	Sparse sampling	LC–MS/MS
7	Kongthavonsakul et al. (2016)	Prospective	2016	Thailand	Intensive sampling	HPLC-UV
8	Pettit et al. (2016)	Prospective	2016	USA	Intensive sampling	HPLC
9	Cies et al. (2017)	Retrospective	2017	USA	Sparse sampling	LC–MS/MS
10	Ohata et al. (2019)	Retrospective	2019	Japan, USA	Sparse sampling	HPLC
11	Wang et al. (2020)	Prospective	2020	China	Sparse sampling	HPLC-UV
12	Saito et al. (2021)	Retrospective	2021	Japan	Sparse sampling	LC–MS/MS
13	Wang Y. et al. (2021)	Prospective	2021	China	Intensive sampling	HPLC-MS/MS
14	Zylbersztajn et al. (2021)	Prospective	2021	Chile, Australia	Sparse sampling	HPLC-UV
15	Yonwises et al. (2021)	Prospective	2021	Thailand	Intensive sampling	HPLC-UV
16	Wu et al. (2022)	Prospective	2022	China	Sparse sampling	HPLC
17	Pokorná et al. (2024)	Retrospective	2024	Czech, Sweden	Sparse sampling	LC–MS/MS
18	Butragueño-Laiseca et al. (2024)	Prospective	2024	Spain	Intensive sampling	HPLC-UV
19	Junior et al. (2024)	Prospective	2024	USA	Sparse sampling	HPLC
20	Le et al. (2025)	Prospective	2025	USA	Intensive sampling	LC-MRM-MS

USA, United States of America; HPLC, high-performance liquid chromatography; HPLC–UV, high-performance liquid chromatography–ultraviolet detection; LC–MS/MS, liquid chromatography–tandem mass spectrometry; HPLC/MS, high-performance liquid chromatography/mass spectrometry; LC-MRM-MS, liquid chromatography multiple reaction monitoring mass spectrometry; NR, no record.

### Characteristics of the included patients

The characteristics of the patients included in each study are shown in Table 2. Pediatric patients with various characteristics were included, and most of them had severe infections or were admitted to the ICU. Four studies focused on patients with organ support, mainly CRKT and ECMO. The sample sizes were usually small, and 15 studies had sample sizes no more than 50. Most studies had plasma concentration points greater than 100, and two studies obtained other body fluid concentrations (urine and cerebrospinal fluid). The age coverage of the studies is additionally shown in Figure 1. Most studies covered a broad range of pediatric patients, and several studies focused on neonates and infants.

**Table 2:** Patient characteristics of each included study.

ID	Author	Patient group	Number of patients	Sample size	Gender (Male/Female)	Age (Year)	Weight (kg)
1	Du et al. (2006)	Pediatric patients with bacterial meningitis or other infections	99 (for PopPK analysis)	425	NR	4.34 ± 3.80, (0.08–17.33)	16.80 ± 11.58, (3.70–65.00)
2	Bradley et al. (2008)	Neonates	37	NR	17/20	GA: 34 ± 5 weeks, (23–41) weeks; PNA: 10 ± 15, (1–60) days	2.4 ± 1.0, (0.8–4.0)
3	Van Den Anker et al. (2009)	Preterm and full-term neonates with suspected/proven bacterial infections	38 neonates (23 preterm, 15 full-term)	390	23/15 (Preterm:13/10, Full-term:10/5)	Preterm: 32 ± 2 weeks, (29–36) weeks; Full-term: 39 ± 1 weeks, (37–42) weeks;	Preterm: 1.87 ± 0.53, (0.952–2.83); Full-term: 3.17 ± 0.47, (2.34–4.05);
4	Ikawa et al. (2010)	Japanese pediatric patients	40	229 plasma samples; 61 urine samples	30/10	6.6 ± 4.1, (0.2–14.8)	23.2 ± 13.5, (3.8–64.0)
5	Ohata et al. (2011)	Japanese pediatric patients with various infectious diseases	50	116	30/20	3.1 ± 3.2, (0–13)	14.8 ± 8.1, (6.5–50.0)
6	Smith et al. (2011)	Infants with suspected or complicated intra-abdominal infections	188 (analyzed out of 200 enrolled)	780 (753 used in final analysis)	NR	GA at birth: 28 (23–40) weeks; PNA at PK evaluation: 21 (1–92) days	NR
7	Kongthavonsakul et al. (2016)	Children with severe infection in the general wards	14	84	9/5	7.1 ± 2.4, 6.0 (4.5–11.8)	22.7 ± 9.7, 20.0 (14.0–46.5)
8	Pettit et al. (2016)	Children with CF	30	120	14/16	12.7 ± 2.9, (8–17)	40.9 ± 12.2, (20.4–68.2)
9	Cies et al. (2017)	Critically ill young children admitted to the PICU	9	16	3/6	3.1 ± 2.9, (1–9)	17.1 ± 11.9, (7.5–40)
10	Ohata et al. (2019)	Pediatric patients with bacterial meningitis	154 (additional 5 adult patients for model development)	Plasma: 207 samples from 141 pediatric patients and 14 samples from five adult patients; CSF: 84 samples from 78 pediatric patients and 14 CSF samples from five adult patients	Study 1: 30/20; Study 2: 79/50; Study 3: 40/32	30.6 ± 34.4 months	12.4 ± 7.6
11	Wang et al. (2020)	Critically ill infants and children with severe infections	57	135	NR	2.96 ± 3.70, 1.44 (0.101–14.4)	15.8 ± 12.6, 11.0 (5.00–65.0)
12	Saito et al. (2021)	Critically ill pediatric patients	34 (8 CRRT, 3 ECMO)	290 (from 380 collected samples)	13/21	1.4 (0.03–14.6)	8.9 (2.7–40.9)
13	Wang Y. et al. (2021)	Septic children receiving ECLS	25 (13 ECMO, 9 CRRT, 4 combined)	225	13/12 (ECLS: 6/7, non-ECLS: 7/5)	2.00 (0.71, 3.88); ECLS: 2.25 (0.69, 4.25); non-ECLS: 1.25 (0.67, 2.50)	11.50 (9.50, 36.30)
14	Zylbersztajn et al. (2021)	Pediatric patients on ECMO support, with 4 on CRRT	9	33	4/5	48 (2–165) months	16 (3.5–45)
15	Yonwises et al. (2021)	Critically ill infants	35	160	16/19	7.53 (1.13–22.50) months	5.70 (3.00–15.02)
16	Wu et al. (2022)	Chinese newborns with LOS	78	110	NR	PNA: 17.6 ± 19.2 days, 12.0 (1.00–113) days; PMA: 37.5 ± 4.40 weeks, 38.2 (27.4–46.1) weeks; GA: 35.0 ± 4.65 weeks, 36.5 (26.3–42.1) weeks	birth weight: 2349 ± 973 g, 2,500 (480–4,100) g; CW: 2661 ± 927 g, 2,680 (980–5,310) g
17	Pokorná et al. (2024)	In neonatal and pediatric ECMO and CRRT	45(38 ECMO, 31 CRRT)	152	23/22	3 (0–465) days	7.88 (3.62–11.97)
18	Butragueño-Laiseca et al. (2024)	Critically ill children	16 critically ill children (7 undergoing CKRT)	212 (118 from patients under CKRT, 94 from patients without CKRT)	NR	CKRT group: 48 (5–106) months; Non-CKRT group: 8 (3.5–82) months	CKRT group: 20 (7.4–40); Non-CKRT group: 7.5 (5.5–17.5)
19	Junior et al. (2024)	Critically ill pediatric and young adult patients in the PICU	48	296	30/18	13.4 (1 month-30 years)	29 (16.2–55.7) *
20	Le et al. (2025)	Critically ill infants (≥4 weeks) and children (<21 years) with sepsis/septic shock admitted to PICU (cases) and nonseptic children (controls)	27(19 septic cases, 8 nonseptic controls)	309	18/9	11.8 (0.6–19.6)	36.3 (7.2–98.0)

The age and weight values are presented as the means ± SDs (ranges) and/or medians (ranges). *The weight values are presented as medians (Q1–Q3).

CSF, cerebrospinal fluid; PK, pharmacokinetics; ECMO, extracorporeal membrane oxygenation; CRRT, continuous renal replacement therapy; CKRT, continuous kidney replacement therapy; LOS, late-onset sepsis; PICU, pediatric intensive care unit; CF, cystic fibrosis; ECLS, extracorporeal life support; GA, gestational age; PMA, gestational age; PNA, postnatal age; CW, current weight; NR, no record.

**Figure 1:**
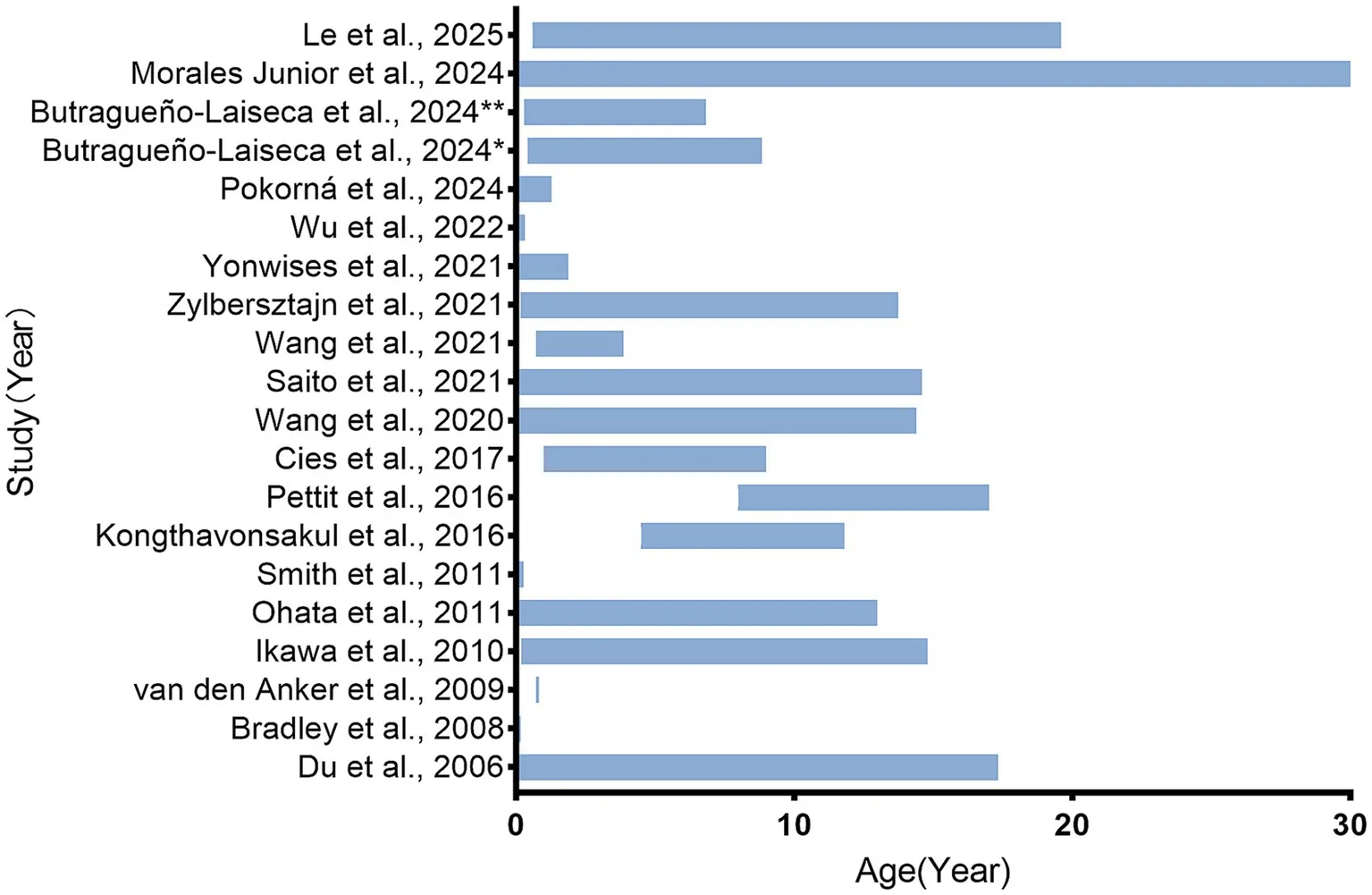
Age coverage of the included studies.

### PPK modeling strategy

The modeling strategies of the included studies are shown in Table 3. NONMEM was the most frequently used modeling software. The covariate screening methods were similar among the studies, with most common forward inclusion criteria of an OFV decrease of 3.84. The exclusion criteria ranged from 3.84 to 10.83. The screened and identified covariates are summarized in Figure 2. Twelve different covariates were incorporated into the final models. In 19 studies, body weight was chosen as a candidate covariate, and in the other study, clearance and V were predominantly scaled by body weight (Carmeli et al., 2025). Finally, two studies failed to include body weight in the final model because of the limited sample size. Renal function was the second most frequently screened, with 18 studies and 11 studies including renal function indicators in the final model. Common renal function indicators included CLCR, Scr, eGFR and other indicators.

**Table 3:** Modeling strategy of the included studies.

ID	Author	Software	Covariate analysis method	Covariates screened	Covariates incorporated
1	Du et al. (2006)	NONMEM version V	Forward inclusion (ΔOBJ >3.84, *p* < 0.05) followed by backward deletion (ΔOBJ >7.88, *p* < 0.005)	Age, WT, BSA, SCr, CLcr, unit body weight dosage (dose/weight)	CLcr, WT on CL; WT on V_c_, V_p_
2	Bradley et al. (2008)	NONMEM version V.1	Forward inclusion and backward exclusion(MOF by>6.6)	GA, PNA, PCA, WT, SCr	SCr, PCA on CL, WT
3	Van Den Anker et al. (2009)	NPAG	Stepwise forward and stepwise backward	Sex, GA, WT, crown-heel length, CLcr, preterm versus full-term status, dose	WT and CLcr on CL
4	Ikawa et al. (2010)	NONMEM 6	Stepwise forward inclusion(ΔOFV>3.84), followed by backward elimination(ΔOFV>6.63)	WT, age	WT on Cl_r_, V_c_, V_p_
5	Ohata et al. (2011)	NONMEM version V	Forward inclusion (ΔOBJ>3.84, *p* < 0.05) followed by backward deletion process (ΔOBJ>6.63, *p* < 0.01)	WT, age, CLcr, SCr	WT on CL, V_c_, Q, V_p_
6	Smith et al. (2011)	NONMEM version VI	Forward inclusion (ΔOFV>3.84, *p* < 0.05) with backward elimination (ΔOFV>7.88, *p* < 0.005)	SCr, PNA, GA, PMA, ALB, sex, UOP, DOPA use	PMA, SCr on CL; ALB on V
7	Kongthavonsakul et al. (2016)	NLME 1.3	A forward inclusion (ΔOFV>6.635) and a backward deletion (ΔOFV>10.828)	Age, WT, BMI, CLcr	None
8	Pettit et al. (2016)	Pmetrics for R (version 1.4.1)	Linear regression followed by incorporation into population model; evaluated by AIC reduction	Age, height, WT, SCr, eGFR, BMI, BSA, lean body weight	WT on CL, V_c_
9	Cies et al. (2017)	Pmetrics	Linear regression followed by re-entry into the model using AIC, likelihood ratio test, visual predictive checks for improvement; allometric and linear scaling were also tested	WT, age, eCLcr	NR
10	Ohata et al. (2019)	NONMEM version 7.3	Objective function and biological and pharmacological plausibility	Age, WT, clinical laboratory data (CSF protein, WBC, glucose)	WT on CL, V_c_, Q, V_p_; Age on k_13_
11	Wang et al. (2020)	NONMEM v7.2.0	Forward inclusion(ΔOFV>3.84, *p* < 0.05) and backward elimination(ΔOFV>6.635, *p* < 0.01)	WT, age, ALT, AST, CLcr, BUN, GGT, ALP, ALB	WT on CL and V_1_ CL_Cr_ on CL
12	Saito et al. (2021)	Phoenix NLME 8.2	Forward inclusion (ΔOFV>3.84, *p* < 0.05) and backward elimination (ΔOFV>7.88, *p* < 0.005)	eGFR, daily urine excretion volume, daily ascites excretion volume, WT, CRRT use, SIRS, proven bacterial infection, ECMO, liver transplantation, immunosuppressant use	eGFR, SIRS, WT on CL WT on V_c_, V_p_, Q CRRT use on V_c_
13	Wang Y. et al. (2021)	NONMEM version 7.4	A stepwise forward additive approach (*p* < 0.05) followed by a stepwise backward elimination approach (*p* = 0.001)	WT, height, age, eCLcr, ECMO intervention, nutrition status (WAZ), liver function, sex, concomitant drugs	WT on CL, V; eCLcr on CL
14	Zylbersztajn et al. (2021)	PMetrics 1.5.0	Sequential assessment of biologically plausible clinical characteristics with linear/nonlinear associations	Age, WT, height, sex, C-reactive protein, ALB, total bilirubin, SCr, 24-h hydric balance	WT on V_c_, K_pc_, K_cp_, CL
15	Yonwises et al. (2021)	NONMEM version 7.4.3	Stepwise forward inclusion (ΔOFV > 3.84, *p* < 0.05) and backward elimination (ΔOFV > 6.64, *p* < 0.01)	Sex, age, WT, CLcr, use of mechanical ventilation, use of vasoactive medications, sepsis or septic shock, congestive heart failure	WT, CLcr on CL WT on V_d_
16	Wu et al. (2022)	NONMEM V7.4	A forward addition (ΔOFV > 3.84) and backward selection (ΔOFV > 6.635)	BW, CW, PNA, GA, PMA, ALB, BUN, Scr	CW, PNA, GA on CL CW on V
17	Pokorná et al. (2024)	NONMEM V7.3.0	Stepwise covariate modeling (ΔOFV > 3.84, *p* < 0.05)	WT, PNA, SCr, eCLcr, serum urea, ALB, bilirubin, liver enzymes, pH, C-reactive protein, PIM3 score, inotropes use, diuretics use, CRRT, ECMO modality, ECMO parameters (flow, speed, duration)	WT on CL and V_d_; CRRT on V_d_
18	Butragueño-Laiseca et al. (2024)	NONMEM 7.4	SCM with forward selection (ΔOFV > 3.84) and backward deletion (ΔOFV > 6.61)	Height, age, WT, ALB, concentration, eGFR, CKRT, hemofilter surface area	eGFR, age on CL_r_; WT on V_1_; Hemofilter surface area on CL_CKRT_
19	Junior et al. (2024)	Monolix 2023R1 Version	Stepwise regression with forward selection (ΔOFV > 3.84, *p* < 0.05) and backward elimination (ΔOFV > 6.64, *p* < 0.01)	WT, eGFR, BUN, daily urine output, blood lactate, age, PRISMIII score, PIM2 score, procalcitonin, vasoactive drug use, *Cum*%FB	WT on CL, V, Q, V_2_; eGFR on CL; *Cum*%FB on V;
20	Le et al. (2025)	NONMEM 7.3	A forward selection (ΔOFV > 4, *p* ≤ 0.05) and a backward elimination (ΔOFV > 8, *p* ≤ 0.005)	WT, eGFR, SCys, SNgal, ALB, complicated appendicitis, Scr	WT, eGFR-MS on CL_MERO_ WT, SCys on V_1-MERO_

BMI, body mass index; CLcr, creatinine clearance; CSF, cerebrospinal fluid; WBC, white blood cell; WT, body weight; WT_i_, individual bodyweight; CL, total body clearance; V_c_, volume of distribution for central compartment; Q, intercompartmental clearance between V_c_ and V_p_; V_p_, volume of distribution for peripheral compartment; V_CSF,_ volume of distribution for CSF compartment; CL_flow_, CSF flow clearance; k_13_, first-order intercompartmental transfer rate constants; OBJ, the minimum value of the NONMEM objective function; OFV, objective function value; V_1_, central volume of distribution; RF, renal function; SCys, serum cystatin C; eGFR, Scr-based estimated glomerular filtration rate; Scr, serum creatinine; CRRT, continuous renal replacement therapy; SIRS, systemic inflammatory response syndrome; ECMO, extracorporeal membrane oxygenation; PNA, postnatal age; PIM3, pediatric index of mortality 3; CRRT_on, binary parameter indicating whether CRRT treatment was on (1) or off (0); SCM, stepwise covariate modeling; CKRT, continuous kidney replacement therapy; CL_r_, renal clearance; CL_CKRT_, CKRT clearance; BW, birth weight; CW, current weight; GA, gestational age; PMA, postmenstrual age; ALB, albumin; BUN, urea nitrogen; ALT, alanine transaminase; AST, aspartate aminotransferase; ALP, alkaline phosphatase; GGT, glutamyl transpeptidase; PRISMIII score, pediatric mortality score; PIM2 score, pediatric mortality score; Hill, Hill coefficient; Cum%FB, cumulative percentage of fluid balance; AIC, Akaike information criterion; CL_0_, clearance; V_C0_, allometric volume of distribution of the central compartment; eCLcr, estimated creatinine clearance; Vw, typical estimate of volume of the central compartment for a total body weight of 20.4 kg; PCA, postconceptional age; UOP, urine output; DOPA, dopamine and epinephrine; BSA, body surface area; WAZ, Weight Adjusted Z scores; CLM, meropenem clearance; CLMERO, meropenem clearance; V1-MERO, meropenem volume of distribution; NR, No Record.

**Figure 2:**
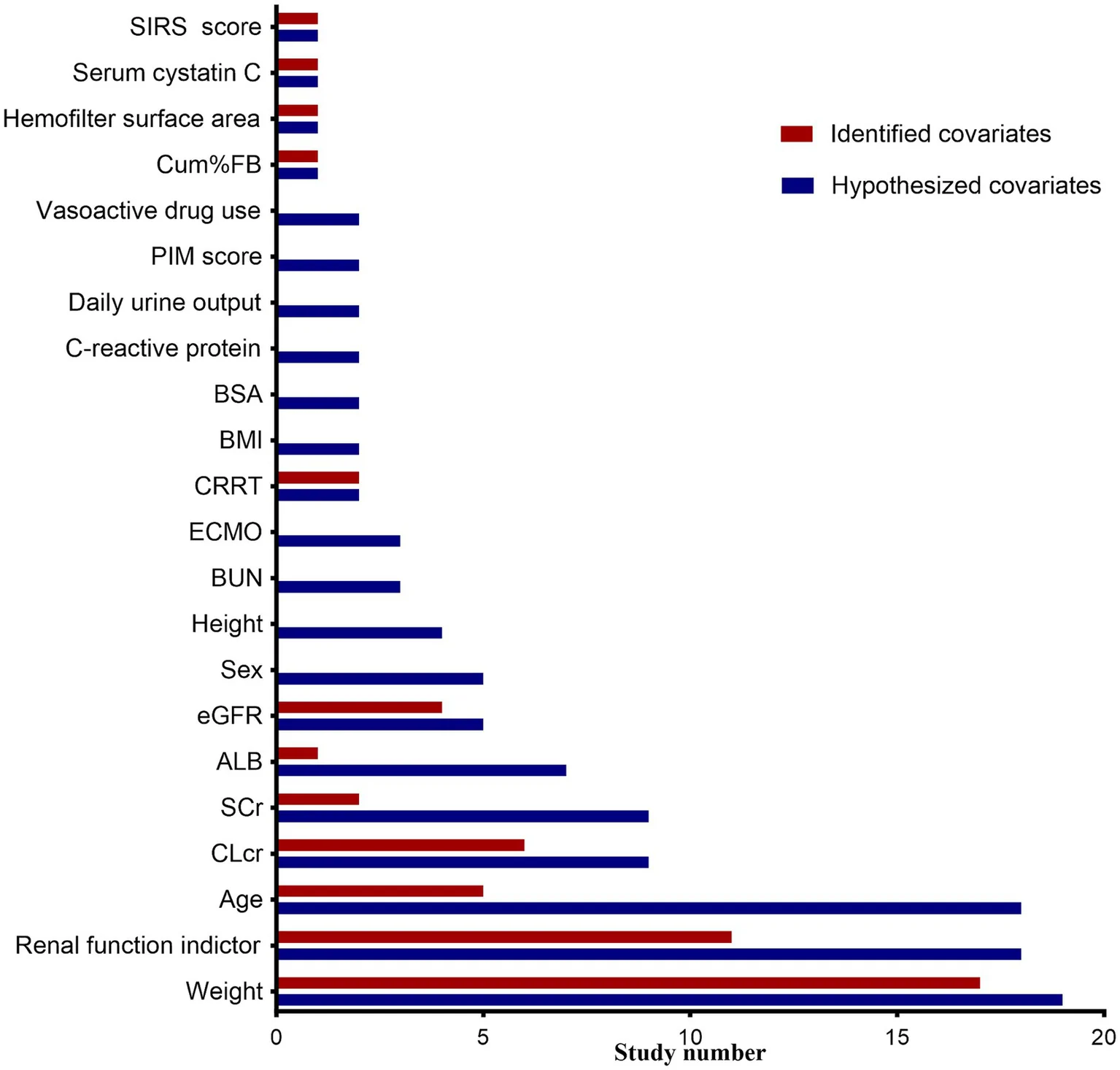
Covariates tested and included in the meropenem population pharmacokinetic model. Covariates tested in a single study but that failed to be included are not shown. CLcr, creatinine clearance; Scr, serum creatinine; ALB, albumin; eGFR, Scr-based estimated glomerular filtration rate; BUN, urea nitrogen; ECMO, extracorporeal membrane oxygenation; CRRT, continuous renal replacement therapy; BMI, body mass index; BSA, body surface area; PIM score, pediatric mortality risk score; *Cum*%FB, cumulative percentage of fluid balance; SIRS, systemic inflammatory response syndrome.

### PPK parameter estimate

The final PPK model information and parameter estimates are shown in Table 4. Fourteen studies (70%) used the final 2-CMT model, 5 studies (25%) used the 1-CMT model, and one study (5%) used the 3-CMT model. The typical CL values are shown in Figure 3, which indicates that variance and heterogeneity existed among the studies. The typical CL values varied greatly among the studies, ranging from 0.50 to 13.22 L/h. The range of weight-scaled CL was also large (0.122–0.526 L/h/kg). The distribution volume of the central compartment (Vc) for the 2-CMT models ranged from 0.969 L to 21.4 L or 0.272 L/kg to 0.57 L/kg. We also stratified the parameters by patient condition, as shown in Supplementary Table S5. Moreover, the interindividual variabilities in parameters were also large in most studies. GOF, bootstrap analysis and VPC were the most common internal validation methods. However, only one study performed external validation.

**Table 4:** Model structure and parameter estimates of the final models.

ID	Author	Compartments	Population typical value	Interindividual variability (IIV)	Residual variability (RV)	Model evaluation method
1	Du et al. (2006)	2-CMT	CL(L/h) = 4.22 × (CLcr/53.35)^0.29^ × (WT/13.5)^0.86^ V_c_(L) = 2.62 × (WT/13.5)^1.06^ Q (L/h) = 2.97 V_p_ (L) = 2.50 × (WT/13.5)^0.30^	CL 30% V_c_ 28% Q 30% V_p_ 48%	Proportional: 18%; Additive: 0.29 mg/L	GOF, bootstrap, MAE, MSE
2	Bradley et al. (2008)	1-CMT	CL (L/h/kg) = 0.041 + 0.040/SCr + 0.003 × (PCA-35) V_d_ (L/kg) = 0.40	CL 14% V_d_ 6%	Proportional: 20%	NR
3	Van Den Anker et al. (2009)	2-CMT	CL (L/h) = 0.156 + 0.00112 × CL_cr_ + 0.0925 × WT V_c_ (L) = 0.969 K_cp_ (h^−1^) = 7.71 K_pc_ (h^−1^) = 30.0	V_c_ 54.5% K_cp_ 86.5% K_pc_ 59.7%	NR	GOF
4	Ikawa et al. (2010)	2-CMT	Cl_r_ (L/h) = 0.254 × WT Cl_nr_ (L/h) = 3.45 V_c_ (L) = 0.272 × WT Q (L/h) = 1.65 V_p_ (L) = 0.228 × WT	Cl_r_ 38.0% Cl_nr_ 139% V_c_ 53.7% Q 104% V_p_ 49.5%	Proportional: 9.87%; Additive: 0.348 μg/mL	Bootstrap, GOF
5	Ohata et al. (2011)	2-CMT	CL (L/h) = 0.428 × WT V_c_ (L) = 0.287 × WT Q (L/h) = 0.0452 × WT V_p_ (L) = 0.0537 × WT	CL 15.2%	σ^2^ = 0.0975 (32.0% CV)	GOF, bootstrap
6	Smith et al. (2011)	1-CMT	CL (L/h/kg) = 0.122 × [(0.5/SCr)^0.267^] × [(PMA/32.7)^1.46^] V (L/kg) = 0.460 × (ALB/2.3)-0.444	CL 30% V 15%	32%	GOF, bootstrap
7	Kongthavonsakul et al. (2016)	2-CMT	V_c_ (L) = 1.93 V_p_ (L) = 2.13 CL (L/h) = 6.53 Q (L/h) = 6.19	V_c_ 77.5% V_p_ 30% CL 34.6%	Additive: 0.16 (0.12–0.17) mg/L	Bootstrap, VPC, GOF
8	Pettit et al. (2016)	2-CMT	CL (L/h/kg) = 0.41 × WT V_c_ (L/kg) = 12.29 × WT/40.9 K_cp_ (h^−1^) = 0.738 K_pc_ (h^−1^) = 1.71	CL 56.1% V_c_ 55.2% K_cp_ 157.9% K_pc_ 93.5%	SD = γ × (0.00761 + 0.0392 × C), γ = 0.755	GOF
9	Cies et al. (2017)	2-CMT	CL (L/h/kg) = 0.419 V_c_ (L/kg) = 0.57 K_cp_ (h^−1^) = 2.512 K_pc_ (h^−1^) = 3.268	CL 35.8% V_c_ 82.5% K_cp_ 57.7% K_pc_ 51.0%	SD = γ × [0.1182 + (0.0179 × C)]	VPCs
10	Ohata et al. (2019)	3-CMT	CL (L/h) = 0.526 × WT V_c_ (L) = 0.292 × WT Q (L/h) = 0.114 × WT V_p_ (L) = 0.108 × WT V_CSF_ (L) = 0.120 k_13_ (h^−1^) = 0.534/1000 × (AGE/14)^0.589^ CL_flow_ (L/h) = 0.021	CL 19.9% k_13_ 204%	Plasma: 56.1%; CSF: 38.8%	Leave-one-out cross validation, VPC
11	Wang et al. (2020)	2-CMT	CL (L/h) = 4.79 × (WT/11)^0.365^ × (CLcr/155.6)^0.317^ V_1_ (L) = 3.68 × (WT/11)^0.435^ V_2_ (L) = 1.67 Q (L/h) = 0.216	CL 12.5% V_1_ 43.4%	Proportional: 38.1%	GOF, bootstrap, NPDE, VPC
12	Saito et al. (2021)	2-CMT	CL (L/h) = 0.46 × (eGFR/38.2)^0.19^ × (1 + 0.35)^SIRS^×WT V_ci_ (L) = 0.49 × (1 + 0.66)^CRRT^×WT V_p_ (L) = 0.34 × WT Q (L/kg/h) = 0.28 × WT	CL 27.10% V_c_ 31.50%	Proportional: 33.23%	GOF, bootstrap, VPC
13	Wang Y. et al. (2021)	2-CMT	CL (L/h) = 7.6 × (WT/12)^0.75^ × [(1 + (eCLcr-150) × 0.0035) + 0.257 × Q × IF CRRT] V_1_ (L) = 21.4 × (WT/12) Q (L/h/kg) = 0.045 × WT SC (%) = 25.7 V_2_ (L) = 0.025	CL 55.7% V_1_ 56.2%	log-transformed concentration Plasma: 0.575 CRRT efflux: 0.284	GOF, VPC, SIR
14	Zylbersztajn et al. (2021)	2-CMT	CL (L/h/kg) = 0.139 V_c_ (L/kg) = 0.289 K_cp_ (h^−1^) = 1.37 K_pc_ (h^−1^) = 1.25	CL 73.3% V_c_ 72.0% K_cp_ 72.6% K_pc_ 79.0%	A multiplicative error model	GOF, VPC
15	Yonwises et al. (2021)	1-CMT	CL (L/h) = 1.33 × exp.(0.126 × (WT-5.70)) + 0.0085 × (CLcr −112.43) V_d_ (L) = 2.27 × exp.(0.139 × (WT-5.70))	CL 28.95% V_d_ 28.02%	Proportional: 28.0%; Additive: 0.463 μg/mL	Bootstrap, GOF, PC-VPC
16	Wu et al. (2022)	1-CMT	CL (L/h) = 1.63 × (CW/2680)^0.75^ × (PNA/12)^0.209^ × (GA/36.5)^2.14^ V (L) = 0.503 × (CW/2680)	V 17.5% CL 50.8%	Proportional: 28.8%	GOF, bootstrap, NPDE, VPC; External validation: MPE, MAPE
17	Pokorná et al. (2024)	1-CMT	CL (L/h) = 1.09 × (WT/7.88) V_d_ (L) = 3.98 × (WT/7.88) × 2.04^CRRT_on^	CL 48% Vd 68%	Proportional: 17%	GOF, NPDE, bootstrap
18	Butragueño-Laiseca et al. (2024)	2-CMT	CL_r_ (L/h) = 5.5 × (eGFR/95) × [AGE/(AGE+15.6)] (eGFR/95) × [AGE/(AGE+15.6)] CL_nr_ (L/h) = 0.91 CL_CKRT_ (L/h) = 1.26 × θ_FILT_, [θ_FILT_MED_ = 1 (reference), θ_FILT_High_ = 1.5, θ_FILT_Low_ = 0.27] V_1_ (L) = 4.75 × WT/10 V_2_ (L) = 10.7 Q(L/h) = 0.28	CL_r_ 30% CL_nr_ 86% V_1_ 48%	Plasma and prefilter: 0.38 log(mg/L) Postfilter: 0.38 log(mg/L) Effluent: 0.37 log(mg/L) Urine: 0.86 log(mg/L)	GOF, PC-VPC, SIRS
19	Junior et al. (2024)	2-CMT	CL (L/h/70 kg^0.75^) = 13.22 × (WT/70)^0.75^ × (eGFR/140)^0.45^ × e^(ηCl + ηIOV)^ × MF; (MF=PMA^Hill^/(PMA^Hill^ + PMA50^Hill^)) V_1_ (L/70 kg) = 16.45 × (WT/70) × e^0.033 × *Cum*%FB^ × e^ηV1 + ηIOV^, Q (L/h/70 kg^0.75^) = 1.78 × (WT/70)^0.75^ V_2_ (L/70 kg) = 7.66 × (WT/70)	IIV: CL 24.3% V_1_ 36.1% IOV: CL 12.0% V_1_ 13.1%	Proportional: 0.33	GOF, VPC, bootstrap
20	Le et al. (2025)	2-CMT	CL (L/h) = 9.72 × (WT/70)^0.75^ × ([eGFR/140])^0.169^ V_1_ (L) = 9.74 × (WT/70) × (1 + 0.449 × SCys/0.45) V_2_ (L) = 4.53 × (WT/70) Q (L/h) = 1.66 × (WT/70)^0.75^	η_V1, V2_ 0.306 η_CL_ 0.487	0.133	GOF, bootstrap

CMT, compartment; CL, clearance; CLcr, creatinine clearance; CL_r_, renal clearance; CL_nr_, nonrenal clearance; CL_0_, clearance; CL_flow_, CSF flow clearance; CL_CKRT_, CKRT clearance; CL_dist_, distributive clearance; V_c_, volume of distribution of the central compartment; _V0_, allometric volume of distribution of the central compartment; V_d_, volume of distribution; Q, intercompartmental clearance between the V_c_ and V_p_; V_CSF_, volume of distribution for the CSF com-partment; CSF, cerebrospinal fluid; V_1_, central volume of distribution; V_2_, peripheral volume of distribution; SC, Sieving coefficient; K_cp_, first-order rate constant for distribution from the central to peripheral compartment; K_pc_, first-order rate constant for distribution from the peripheral to central compartment; k_13_, first-order intercompartmental transfer rate constants; WT, body weight; CW, current weight; CRRT, continuous renal replacement therapy; PMA, postmenopausal age; SCR, serum creatinine; IIV, interindividual variability; IOV, intermediate-occasion variability; GOF, goodness–of-fit plots; MAE, mean absolute error; MSE, mean squared error; VPC, visual predictive checks; PC-VPC, Prediction-Corrected Visual Predictive Check; SIR, sampling importance resampling; NPDE, normalized prediction distribution errors; MPE, mean prediction error; MAPE, mean absolute prediction error; NR, no record.

**Figure 3:**
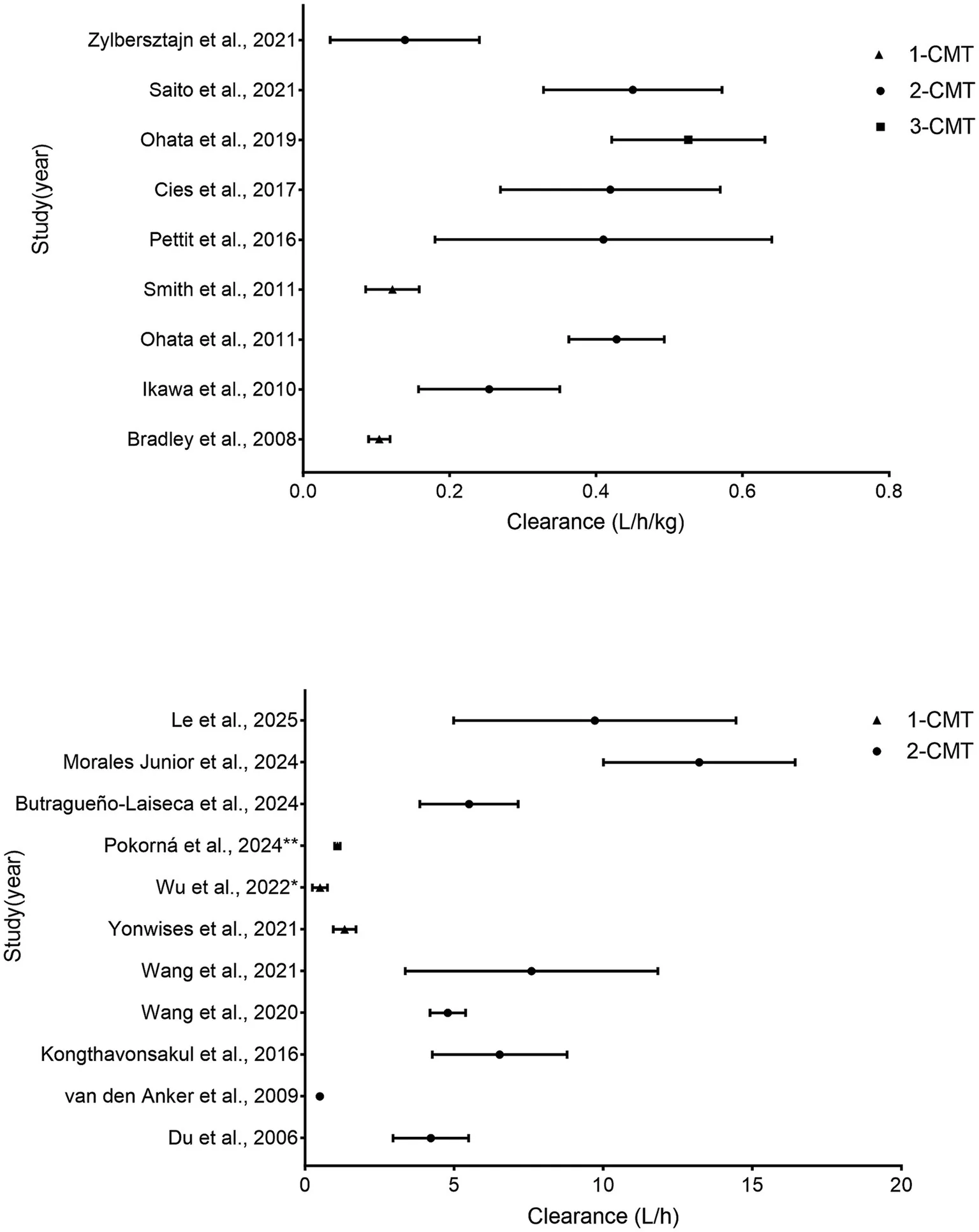
Clearance estimates of the models.*For a typical patient with a CW = 2,680 g. **For a typical patient (7.88 kg, off CRRT).

### Covariate effect

The effect of the covariate on CL was evaluated, and the results are shown in Figure 4. The covariate with the greatest influence on CL was body weight, and the CL may change several-fold with different body weights. Renal function and age had a weaker influence on CL, which was not significant in some studies.

**Figure 4:**
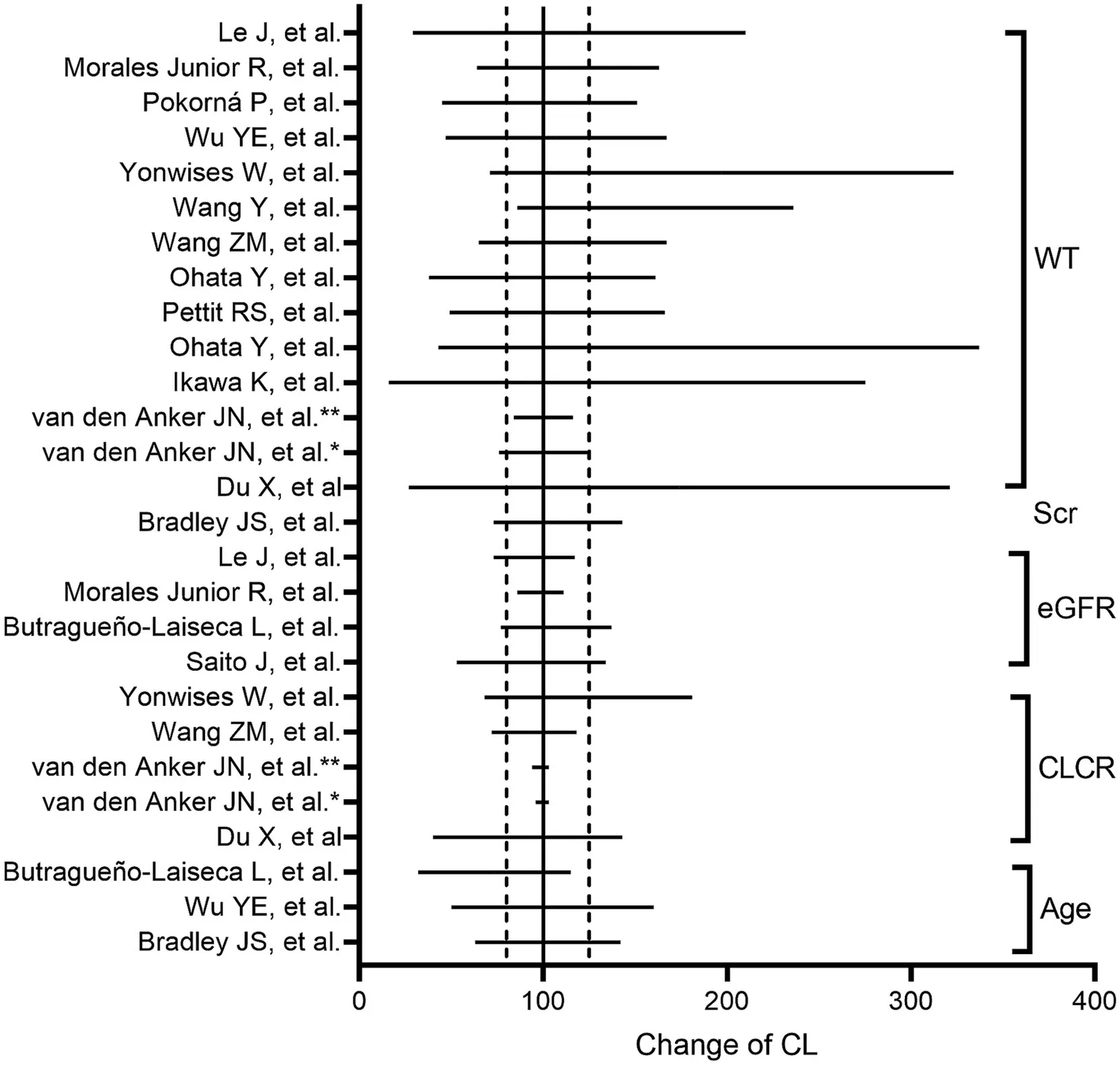
Covariate effect on drug clearance. CL, drug clearance; WT, body weight; CLCR, creatinine clearance rate; eGFR, estimated glomerular filtration rate; Scr, serum creatinine. The effect of CRRT on CL estimated by Wang Y. et al. (2021) is less than 2% and is not shown in the figure.

### Dosage regimen suggestion

In terms of the results of the PK/PD analysis, %T > MIC was the common index. However, the target value varied across studies. Most studies have performed simulations and suggested optimized dosage regimens for specific patient populations (Table 5). The current meropenem dosage may be sufficient for the normal target (40–50%T > MIC), but a higher dose and prolonged infusion are needed for aggressive targets. Several studies have reported the actual PK/PD target attainment. Du et al. reported %T > MIC values ranging from 72% to 100% for an MIC distribution range of 0.004 to 0.6 μg/mL, and 50%T > 4*MIC attainment rates reported by Smith et al. and Zylbersztajn et al. were also satisfactory (Du et al., 2006; Smith et al., 2011; Zylbersztajn et al., 2021).

**Table 5:** PK/PD target for simulation and dose regimen suggestions.

ID	Author	PK/PD target in simulation	Dose regimen suggestion
1	Du et al. (2006)	NA	40 mg/kg dosing regimen provides sufficient meropenem exposures to ensure the eradication of the common causative pathogens in pediatric meningitis.
2	Bradley et al. (2008)	60% T > MIC	10 mg/kg every 12 h for non-CNS infections caused by group B streptococcus and enteric gram-negative bacilli, and 20 mg/kg every 8 h for *P. aeruginosa* requires evaluation for both safety and efficacy in prospective clinical studies in the neonate.
3	Van Den Anker et al. (2009)	40% T > MIC	20- and 40-mg/kg doses 8-h dosing for neonates. If MIC of 4 to 8 mg/liter, the 40-mg/kg dose and a prolonged infusion was favored.
4	Ikawa et al. (2010)	40% T > MIC	the approved regimens of 10–40 mg/kg t.i.d. (0.5-h infusions) had sufficient bactericidal effects against common bacteria in most typical patients (PTA > 80%).
5	Ohata et al. (2011)	50% T > MIC	20 mg/kg t.i.d. with 0.5-h infusion was effective for bacteria with low MICs; 40 mg/kg t.i.d. with a longer infusion duration was suggested for bacteria with MIC≥ 2 μg/mL.
6	Smith et al. (2011)	NA	infants aged <91 days: 20 mg/kg every 12 h in infants <32 weeks GA and PNA < 14 days; 20 mg/kg every 8 h in infants <32 weeks GA and PNA ≥ 14 days and in infants ≥32 weeks GA and PNA < 14 days; and 30 mg/kg every 8 h in infants ≥32 weeks GA and PNA ≥ 14 days
7	Kongthavonsakul et al. (2016)	40% T > MIC	3-h infusion of 20 mg/kg of meropenem every 8-h for sepsis children
8	Pettit et al. (2016)	40% T > MIC	children with cystic fibrosis, 40 mg/kg every 8-h via 3-h infusion
9	Cies et al. (2017)	40% T > MIC and 80% T > MIC	20–40 mg/kg per dose every 8 h via 3- to 4-h prolonged infusion or 24-h continuous infusion for critically ill children ages 1 to 9 years
10	Ohata et al. (2019)	50% T > MIC	A high dose of meropenem (40 mg/kg q8h) is necessary to achieve clinical efficacy in pediatric patients with bacterial meningitis.
11	Wang et al. (2020)	70% T > MIC	For critically ill infants and children, 40 mg/kg/dose (q8h) with 4-h infusion is suggested for bacteria with MIC≤2 μg/mL.
12	Saito et al. (2021)	40% T > MIC and 100% T > MIC	40 to 80 mg/kg every 8 h with a prolonged 3-h infusion for Critically Ill Pediatric Patients to achieve 100% T > MIC
13	Wang Y. et al. (2021)	50% T > MIC and 100% T > MIC	Dosing 20 mg/kg q8h for 1 h infusion (for a target defined as 50% time > MIC, PTA > 70%) is suggested for ECMO pediatric patients (MIC < 1 mg/L). Higher dose is required for higher target or higher MIC.
14	Zylbersztajn et al. (2021)	NA	NA
15	Yonwises et al. (2021)	40% T > MIC and 80% T > MIC	60 mg/kg every 8-h via 1-h infusion is sufficient for common PK/PD target but no aggressive target in critical ill infants.
16	Wu et al. (2022)	70% T > MIC	For LOS with a MIC of 8 mg/L, the doses of 30 mg/kg every 8-h as a 1-h infusion for newborns with GA ≤ 37 weeks and 40 mg/kg every 8-h as a 3-h infusion for those with GA > 37 weeks were optimal, with PTA of 71.71% and 75.08%, respectively.
17	Pokorná et al. (2024)	40% T > MIC and 100% T > MIC	20–40 mg/kg every 8-h provide acceptably high PTA for MIC ≤ 4 mg/L for 40% T > MIC, but individual dose adjustments are needed for 100% T > MIC for neonatal and pediatric ECMO and CRRT patients
18	Butragueño-Laiseca et al. (2024)	90% T > MIC	In pediatric patients undergoing CKRT, keeping the dose to 40 mg/kg q8h without applying renal adjustment and the extended infusions (40 mg/kg 3 or 4-h infusion every 12 h) was sufficient to reach 90% T > MIC (for an MIC of 2 mg/L) in patients > 10 kg. In patients <10 kg, only continuous infusions reached the objective. In patients >30 kg, a 60 mg/kg/24-h infusion is sufficient and avoids accumulation.
19	Junior et al. (2024)	40% T > MIC, 100% T > MIC and 100% T > 4*MIC	20 mg/kg every 8 h with a 3 h infusion for 40% fT > MIC. If more stringent PD targets are considered, continuous infusion regimens ensure optimal target attainment for most patients, ranging from 30 mg/kg/day to 120 mg/kg/day.
20	Le et al. (2025)	NR	NR

PK/PD, pharmacokinetic/pharmacodynamic; T > MIC, time fraction of meropenem plasma level over minimum inhibitory concentration.

## Discussion

This systematic review comprehensively analyzed the available meropenem PPK models for pediatric patients. Most studies have yielded 2-CMT models, with body weight and renal function as the most common covariates. The parameters varied greatly among studies, and the interindividual variabilities within studies were also large, which underscores the importance of MIPD and therapeutic drug monitoring (TDM) for these patients. Among the 20 available models, diverse pediatric patients were included, which would be helpful for meropenem MIPD in pediatric patients.

The 20 included studies were distributed on average from 2006 to 2025, which indicated that the precision dosing of meropenem for pediatric patients was always noted. The prospective design of 14 studies would be helpful for controlling for retrospective bias. Four studies were multicenter studies and were more confident in extrapolating the results. However, only 11 countries were included in the included studies. Although no evidence supporting ethic differences exists, more data from other areas are needed, especially Africa and South America (Boutzoukas et al., 2025). Moreover, differences in clinical practice, dosing, and population characteristics may still affect PK.

In terms of patient characteristics, a variety of patients were included. Pharmacokinetics in special populations, including newborns, neonates, young children, and pediatric patients with critical illness or sepsis, were investigated. When dosing pediatric patients with meropenem, physicians and pharmacists can select a PPK model with characteristics similar to those of a specific patient to improve precision dosing. Some models cover a broad age range, but caution should be taken when these models are applied to newborns because of the limited data of this subset. Organ support could alter the pharmacokinetics of drugs and increase dosing complexity (Stitt et al., 2024; Stitt et al., 2022). Pediatric patients who received organ support were included, mainly those who received CRRT and ECMO. However, fewer than 100 patients were treated with CRRT, and the number of patients treated with ECMO was even lower. The PK of meropenem in these patients still needs more data.

Although various modeling software programs have been used, NONMEM is the most frequently used software, as it is the gold standard in PPK modeling (Duong and Marsot, 2024). The modeling strategies among studies were similar, but the significance levels in backward elimination were different. This may be due to the sample size used for modeling. However, overfitting may exist in studies with small sample sizes. 2-CMT was the predominant model structure among all the studies. The good design of the sampling strategy allowed the studies to develop a more complicated model. Although only 7 studies applied intensive sampling, the other studies used a sparse sampling strategy, but the time points covered the dosing interval. The only 3-CMT model study reported a 2-CMT model, as it classified CSF as a single compartment (Ohata et al., 2019). Thus, the 2-CMT model would be more suitable for pediatric patients, but the complexity may cause difficulty in parameter estimation and model application. Regrettably, none of the studies reported condition numbers or shrinkages. The estimated typical CL value of meropenem ranged from 0.50 to 13.22 L/h, and the Vc for the 2-CMT model ranged from 0.969 L to 21.4 L. This is not surprising because of the physiological difference between neonates and young adolescents (Wang K. et al., 2021). Some reports have scaled the CL by body weight, but the CLs ranged from 0.122 to 0.526 L/h/kg, and the variability was large. These findings indicated that TDM is important for obtaining optimal meropenem exposure in pediatric patients (Luque et al., 2024).

Many covariates were tested, and 11 different covariates were incorporated into the final models. These covariates can be divided into two main groups. The first group was the covariate that indicated the growth status of the pediatric patients, mainly body weight and age. Nearly all the studies tested these two variables; 17 studies included body weight (an additional study predominantly included body weight), and 11 studies included age. In pediatric patients with severe disease, body weight is a better indicator of growth status, especially in neonates and infants (Soejima et al., 2022). This is in accordance with the label suggestion of weight-scaled dosing. However, the PK of meropenem in obese and malnourished children was not assessed, and caution should be taken when weight-scaled dosing is used for these patients. The second group was the covariate that indicated renal function. It is quite reasonable that meropenem is eliminated mainly through the renal pathway and that decreased renal function can reduce drug clearance (Soejima et al., 2022). There are many renal function indicators, and the indicators included in these studies include CRCL, eGFR, SCr, CRRT, serum cystatin C, etc. For creatine clearance or glomerular filtration rate estimation, multiple functions were used, and Schwartz and modified Schwartz functions were most frequently used. However, these functions are limited in some subgroups, such as patients aged under 1 year, patients with AKI, and malnourished patients (Kudgus Lokken et al., 2026). It should be noted when these patients are treated with covariates. Other kinds of covariates have seldom been tested. Body weight, age and renal function are insufficient to explain the variability in meropenem PK among individuals.

Among all the included studies, multiple internal model validation methods were applied, but only one model underwent external validation. This may be due to the lack of available datasets and methodological challenges. The common internal validation methods included goodness-of-fit (GOF) plots, visual predictive checks (VPCs), and the bootstrap method. One study performed leave-one-out cross-validation, but it was also internally validated, as the datasets for model development and validation were the same. Each internal validation method has shortcomings. The GOF method is intuitive and fast, but it is highly subjective and lacks quantitative indicators. The VPC method is more suitable for evaluating the overall performance of a model than for estimating the accuracy of the parameters. The bootstrap method cannot be used to evaluate the predictive performance of the model. A combination of multiple internal validation methods would improve the robustness of the model. However, the extrapolation performances of these models are uncertain because of the lack of external validation. When these models are applied, it is necessary to carefully select the model. Clinicians should compare patient characteristics (weight, renal function, and other covariates) with those of patients for model development and select the model with least extrapolation risk. However, common modeling tools, such as NONMEM and Monolix, are difficult to handle, and a pharmacist would be helpful. It is also better to perform local validation or re-estimation of parameters before model application; usually, a dataset of 10–20 patients is needed for external validation (Sherwin et al., 2012; El Hassani et al., 2025). Owing to the large variability in pediatric meropenem PK, TDM is the most reliable method to achieve optimal exposure at the current stage. More accurate dose adjustments could be performed using Bayesian forecasting based on the PPK model and TDM data. When TDM data accumulate, clinicians can practice MIPD, and treatment outcomes may improve along with improved target achievement.

Most studies have performed simulations based on the final PPK model and have provided dosing suggestions. Body weight and renal function are the main patient issues that should be considered when meropenem is given in pediatric patients. The common dose of 20–40 mg/kg every 8-h regimen could achieve 40% T > MIC in most circumstances. However, when the infected microorganism is less susceptible or an aggressive PK/PD target is needed, a higher dose and prolonged infusion should be selected. However, an aggressive dose may be off-label, and the risk and benefit should be evaluated carefully. According to the covariate analysis, body weight has the greatest influence on CL, and a weight-scale dose is preferred. This is in accordance with the simulation data. Renal function influenced CL, but the effect was not significant under some circumstances. Approximately 50% of the dose adjustment may be required for AKI and augmented renal function patients.

Future studies are still needed to address the current limitations of the available models. Well-designed studies on meropenem PK in diverse pediatric patients with specific pathophysiological conditions are needed. Currently, the PK in neonates, patients with CRRT and ECMO support, patients with malnutrition and patients with organ dysfunction is insufficient. Novel covariates should be identified to describe the intraindividual variability. Multicenter external validation of available PPK models or development of a universal PPK model is needed to evaluate the external performance. Advanced models, such as the PBPK model and artificial intelligence technology, are needed to explore the origin of individual variability.

## Conclusion

This systematic review successfully integrated the current knowledge about PPK models for pediatric patients. The available PPK models cover diverse pediatric patients, and body weight, age and renal function are the most important covariates. The synthesized evidence provides a valuable reference for future PPK model development and supports individualized meropenem dosing in pediatric clinical practice. Considering the limitations of the current models, multicenter external validation and future well-designed studies in diverse pediatric populations (neonates, septic patients, patients with CRRT and ECMO, etc.) are needed. Clinical decision support tools for precision dosing of meropenem in pediatric patients would be favored.

## Data Availability

The original contributions presented in the study are included in the article/Supplementary material, further inquiries can be directed to the corresponding authors.

## References

[ref1] Alikhani M. S. Nazari M. Hatamkhani S. (2025). Enhancing antibiotic therapy through comprehensive pharmacokinetic/pharmacodynamic principles. Front. Cell. Infect. Microbiol. 15:1521091. doi: 10.3389/fcimb.2025.1521091, 40070375 PMC11893874

[ref2] Balkhy H. H. El-Saed A. AlShehri A. Alshaalan M. Hijazi O. El-Metwally A. . (2019). Antimicrobial consumption in three pediatric and neonatal intensive care units in Saudi Arabia: 33-month surveillance study. Ann. Clin. Microbiol. Antimicrob. 18:20. doi: 10.1186/s12941-019-0320-2, 31269955 PMC6607581

[ref3] Boutzoukas A. E. Balevic S. J. Hemmersbach-Miller M. Winokur P. L. Gu K. Chan A. W. . (2025). Population pharmacokinetics of meropenem across the adult lifespan. Clin. Pharmacokinet. 64, 229–241. doi: 10.1007/s40262-024-01465-1, 39681779 PMC12181987

[ref4] Bradley J. S. Harvey H. Stout D. Momper J. Capparelli E. Avedissian S. N. . (2025). Subtherapeutic meropenem antibiotic exposure in children with septic shock assessed by noncompartmental pharmacokinetic analysis in a prospective dataset. Pediatr. Crit. Care Med. 26, e507–e515. doi: 10.1097/PCC.0000000000003698, 39964222 PMC11960678

[ref5] Bradley J. S. Sauberan J. B. Ambrose P. G. Bhavnani S. M. Rasmussen M. R. Capparelli E. V. (2008). Meropenem pharmacokinetics, pharmacodynamics, and Monte Carlo simulation in the neonate. Pediatr. Infect. Dis. J. 27, 794–799. doi: 10.1097/INF.0b013e318170f8d2, 18645546

[ref6] Butragueño-Laiseca L. Troconiz I. F. Grau S. Campillo N. Padilla B. Fernández S. N. . (2024). How to use meropenem in pediatric patients undergoing CKRT? Integrated meropenem pharmacokinetic model for critically ill children. Antimicrob. Agents Chemother. 68:e0172923. doi: 10.1128/aac.01729-23, 38656186 PMC11620509

[ref7] Carmeli Y. Cisneros J. M. Paul M. Daikos G. L. Wang M. Torre-Cisneros J. . (2025). Aztreonam-avibactam versus meropenem for the treatment of serious infections caused by gram-negative bacteria (REVISIT): a descriptive, multinational, open-label, phase 3, randomised trial. Lancet Infect. Dis. 25, 218–230. doi: 10.1016/S1473-3099(24)00499-7, 39389071

[ref8] Cies J. J. Moore W. S. Enache A. Chopra A. (2017). Population pharmacokinetics and pharmacodynamic target attainment of meropenem in critically ill young children. J. Pediatr. Pharmacol. Ther. 22, 276–285. doi: 10.5863/1551-6776-22.4.276, 28943823 PMC5562208

[ref9] Du X. Li C. Kuti J. L. Nightingale C. H. Nicolau D. P. (2006). Population pharmacokinetics and pharmacodynamics of meropenem in pediatric patients. J. Clin. Pharmacol. 46, 69–75. doi: 10.1177/0091270005283283, 16397286

[ref10] Duong A. Marsot A. (2024). Nlmixr2 versus NONMEM: An evaluation of maximum a posteriori Bayesian estimates following external evaluation of gentamicin and tobramycin population pharmacokinetic models. Clin. Pharmacol. drug Dev. 13, 739–747. doi: 10.1002/cpdd.1395, 38465725

[ref11] El Hassani M. Thirion D. J. G. Marsot A. (2025). Population pharmacokinetic model evaluation with a small real-world dataset versus a large virtual dataset: does sample size affect decision-making? Eur. J. Drug Metab. Pharmacokinet. 50, 441–445. doi: 10.1007/s13318-025-00960-5, 40730936

[ref12] Goulooze S. C. Vis P. W. Krekels E. H. J. Knibbe C. A. J. (2023). Advances in pharmacokinetic-pharmacodynamic modelling for pediatric drug development: extrapolations and exposure-response analyses. Expert. Rev. Clin. Pharmacol. 16, 1201–1209. doi: 10.1080/17512433.2023.2288171 38069812

[ref13] Guo Y. Han R. Jiang B. Ding L. Yang F. Zheng B. . (2022). In vitro activity of new β-lactam-β-lactamase inhibitor combinations and comparators against clinical isolates of gram-negative bacilli: results from the China antimicrobial surveillance network (CHINET) in 2019. Microbiol. Spectrum 10:e0185422. doi: 10.1128/spectrum.01854-22 PMC9431184 35862963

[ref14] Ikawa K. Morikawa N. Ikeda K. Miki M. Kobayashi M. (2010). Population pharmacokinetics and pharmacodynamics of meropenem in Japanese pediatric patients. J. Infect. Chemother. 16, 139–143. doi: 10.1007/s10156-009-0025-0, 20094749

[ref15] Jamsen K. M. McLeay S. C. Barras M. A. Green B. (2014). Reporting a population pharmacokinetic–pharmacodynamic study: a journal’s perspective. Clin. Pharmacokinet. 53, 111–122. doi: 10.1007/s40262-013-0114-1 24327237

[ref16] Junior R. M. Mizuno T. Paice K. M. Pavia K. E. Hambrick H. R. Tang P. . (2024). Identifying optimal dosing strategies for meropenem in the paediatric intensive care unit through modelling and simulation. J. Antimicrob. Chemother. 79, 2668–2677. doi: 10.1093/jac/dkae274, 39092928 PMC11442002

[ref17] Kanji S. Hayes M. Ling A. Shamseer L. Chant C. Edwards D. J. . (2015). Reporting guidelines for clinical pharmacokinetic studies: the ClinPK statement. Clin. Pharmacokinet. 54, 783–795. doi: 10.1007/s40262-015-0236-8, 25637173

[ref18] Kongthavonsakul K. Lucksiri A. Eakanunkul S. (2016). Pharmacokinetics and pharmacodynamics of meropenem in children with severe infection. Int. J. Antimicrob. Agents 48, 151–157. doi: 10.1016/j.ijantimicag.2016.04.025, 27345269

[ref19] Kudgus Lokken R. A. Illamola S. M. Job K. M. Al-Sallami H. S. Jong G. W. Reith D. M. . (2026). Advancing pediatric dose scaling: strategies, modeling approaches, and clinical applications. Pharmaceuticals 19:1090. doi: 10.3390/ph19071090, 42515770 PMC13415250

[ref20] Le J. Huynh J. Vo B. Mai A. Mak R. H. Momper J. D. . (2025). Variability in meropenem distribution and clearance in children with sepsis: population - based pharmacokinetics with assessment of renal biomarkers. Clin. Pharmacokinet. 64, 769–777. doi: 10.1007/s40262-025-01495-3, 40272699 PMC12064575

[ref21] Luque S. Mendoza-Palomar N. Aguilera-Alonso D. Garrido B. Miarons M. Piqueras A. I. . (2024). Therapeutic drug monitoring of antibiotic and antifungical drugs in paediatric and newborn patients. Consensus guidelines of the Spanish Society of Hospital Pharmacy (SEFH) and the Spanish Society of Paediatric Infectious Diseases (SEIP). Anales de Pediatría (English Edition) 101, 190–207. doi: 10.1016/j.anpede.2024.05.001, 39278690

[ref22] Martin-Loeches I. Timsit J.-F. Kollef M. H. Wunderink R. G. Shime N. Nováček M. . (2022). Clinical and microbiological outcomes, by causative pathogen, in the ASPECT-NP randomized, controlled, phase 3 trial comparing ceftolozane/tazobactam and meropenem for treatment of hospital-acquired/ventilator-associated bacterial pneumonia. J. Antimicrob. Chemother. 77, 1166–1177. doi: 10.1093/jac/dkab494, 35022730 PMC9432134

[ref23] Meschiari M. Asquier-Khati A. Tiseo G. Luque-Paz D. Murri R. Boutoille D. . (2024). Treatment of infections caused by multidrug-resistant gram-negative bacilli: a practical approach by the Italian (SIMIT) and French (SPILF) societies of infectious diseases. Int. J. Antimicrob. Agents 64:107186. doi: 10.1016/j.ijantimicag.2024.107186 38688353

[ref24] Mu Z. Wang J. Mu E. (2025). Impact of prolonged versus intermittent infusion of meropenem on mortality and clinical outcomes in patients with severe infection: a systematic review and meta-analysis. J. Infect. Chemother. 31:102634. doi: 10.1016/j.jiac.2025.102634, 39864658

[ref25] Ohata Y. Tomita Y. Nakayama M. Kozuki T. Sunakawa K. Tanigawara Y. (2011). Optimal dosage regimen of meropenem for pediatric patients based on pharmacokinetic/pharmacodynamic considerations. Drug Metab. Pharmacokinet. 26, 523–531. doi: 10.2133/dmpk.DMPK-11-RG-027, 21747200

[ref26] Ohata Y. Tomita Y. Sunakawa K. Drusano G. L. Tanigawara Y. (2019). Cerebrospinal pharmacokinetic and pharmacodynamic analysis of efficacy of meropenem in paediatric patients with bacterial meningitis. Int. J. Antimicrob. Agents 54, 292–300. doi: 10.1016/j.ijantimicag.2019.06.021, 31279154

[ref27] Perez F. El Chakhtoura N. G. Bonomo R. A. (2025). Management of Severe Infections: multidrug-resistant and carbapenem-resistant gram-negative Bacteria. Med. Clin. North Am. 109, 735–747. doi: 10.1016/j.mcna.2025.01.003, 40185559

[ref28] Pettit R. S. Neu N. Cies J. J. Lapin C. Muhlebach M. S. Novak K. J. . (2016). Population pharmacokinetics of meropenem administered as a prolonged infusion in children with cystic fibrosis. J. Antimicrob. Chemother. 71, 189–195. doi: 10.1093/jac/dkv289, 26416780

[ref29] Pokorná P. Michaličková D. Tibboel D. Berner J. (2024). Meropenem disposition in neonatal and Pediatric extracorporeal membrane oxygenation and continuous renal replacement therapy. Antibiotics 13:419. doi: 10.3390/antibiotics13050419, 38786147 PMC11117356

[ref30] Rao G. G. Landersdorfer C. B. (2021). Antibiotic pharmacokinetic/pharmacodynamic modelling: MIC, pharmacodynamic indices and beyond. Int. J. Antimicrob. Agents 58:106368. doi: 10.1016/j.ijantimicag.2021.106368, 34058336

[ref31] Roberts J. A. Paul S. K. Akova M. Bassetti M. De Waele J. J. Dimopoulos G. . (2014). DALI: defining antibiotic levels in intensive care unit patients: are current β-lactam antibiotic doses sufficient for critically ill patients? Clin. Infect. Dis. 58, 1072–1083. doi: 10.1093/cid/ciu027, 24429437

[ref32] Saito J. Shoji K. Oho Y. Kato H. Matsumoto S. Aoki S. . (2021). Population pharmacokinetics and pharmacodynamics of meropenem in critically ill Pediatric patients. Antimicrob. Agents Chemother. 65, e01909–e01920. doi: 10.1128/AAC.01909-20, 33199385 PMC7848983

[ref33] Salmon-Rousseau A. Martins C. Blot M. Buisson M. Mahy S. Chavanet P. . (2020). Comparative review of imipenem/cilastatin versus meropenem. Med. Mal. Infect. 50, 316–322. doi: 10.1016/j.medmal.2020.01.001, 32035719

[ref34] Sherwin C. M. T. Kiang T. K. L. Spigarelli M. G. Ensom M. H. H. (2012). Fundamentals of population pharmacokinetic modelling: validation methods. Clin. Pharmacokinet. 51, 573–590. doi: 10.1007/BF03261932, 22799590

[ref35] Smith P. B. Cohen-Wolkowiez M. Castro L. M. Poindexter B. Bidegain M. Weitkamp J. H. . (2011). Population pharmacokinetics of meropenem in plasma and cerebrospinal fluid of infants with suspected or complicated intra-abdominal infections. Pediatr. Infect. Dis. J. 30, 844–849. doi: 10.1097/INF.0b013e31822e8b0b, 21829139 PMC3173561

[ref36] Soejima K. Sato H. Hisaka A. (2022). Age-related change in hepatic clearance inferred from multiple population pharmacokinetic studies: comparison with renal clearance and their associations with organ weight and blood flow. Clin. Pharmacokinet. 61, 295–305. doi: 10.1007/s40262-021-01069-z, 34514537

[ref37] Stitt G. Dubinsky S. Edginton A. Huang Y.-S. V. Zuppa A. F. Watt K. . (2022). Antimicrobial dosing recommendations in Pediatric continuous renal replacement therapy: a critical appraisal of current evidence. Front. Pediatr. 10:889958. doi: 10.3389/fped.2022.889958, 35633961 PMC9134108

[ref38] Stitt G. Thibault C. Mueller B. A. Cies J. J. Daniel J. M. Arikan A. A. . (2024). Pharmacokinetic research in Pediatric extracorporeal therapies: current state and future directions. Blood Purif. 53, 520–526. doi: 10.1159/000534828, 39363977 PMC11449264

[ref39] Tamma P. D. Aitken S. L. Bonomo R. A. Mathers A. J. van Duin D. Clancy C. J. (2022). Infectious Diseases Society of America 2022 guidance on the treatment of extended-spectrum β-lactamase producing Enterobacterales (ESBL-E), carbapenem-resistant Enterobacterales (CRE), and *Pseudomonas aeruginosa* with difficult-to-treat resistance (DTR-P). Clin. Infect. Dis. 75, 187–212. doi: 10.1093/cid/ciac268 PMC9890506 35439291

[ref40] Tan X. Li Y. Xi J. Guo S. Su H. Chen X. . (2021). Comparative efficacy and safety of antipseudomonal β-lactams for pediatric febrile neutropenia: a systematic review and Bayesian network meta-analysis. Medicine (Baltimore) 100:e27266. doi: 10.1097/MD.0000000000027266, 34918626 PMC8678002

[ref41] Tang Girdwood S. Morales Junior R. Schuler E. Tang P. Diseroad E. Misch C. . (2025). Building a Beta-lactam model-informed precision dosing Service in a Quaternary Care Children’s hospital. Clin. Transl. Sci. 18:e70438. doi: 10.1111/cts.70438, 41319194 PMC12665186

[ref42] Uster D. W. Stocker S. L. Carland J. E. Brett J. Marriott D. J. E. Day R. O. . (2021). A model averaging/selection approach improves the predictive performance of model-informed precision dosing: vancomycin as a case study. Clin. Pharmacol. Ther. 109, 175–183. doi: 10.1002/cpt.2065, 32996120

[ref43] Van Den Anker J. N. Pokorna P. Kinzig-Schippers M. Martinkova J. De Groot R. Drusano G. L. . (2009). Meropenem pharmacokinetics in the newborn. Antimicrob. Agents Chemother. 53, 3871–3879. doi: 10.1128/AAC.00351-09, 19581463 PMC2737888

[ref44] Wang Z. M. Chen X. Y. Bi J. Wang M. Y. Xu B. P. Tang B. H. . (2020). Reappraisal of the optimal dose of meropenem in critically ill infants and children: a developmental pharmacokinetic- pharmacodynamic analysis. Antimicrob. Agents Chemother. 64:e00760-20. doi: 10.1128/AAC.00760-20, 32513801 PMC7526843

[ref45] Wang Y. Chen W. Huang Y. Wang G. Li Z. Yan G. . (2021). Optimized dosing regimens of meropenem in septic children receiving extracorporeal life support. Front. Pharmacol. 12:699191. doi: 10.3389/fphar.2021.699191, 34504424 PMC8421735

[ref46] Wang K. Jiang K. Wei X. Li Y. Wang T. Song Y. (2021). Physiologically based pharmacokinetic models are effective support for Pediatric drug development. AAPS PharmSciTech 22:208. doi: 10.1208/s12249-021-02076-w, 34312742 PMC8312709

[ref47] Wu Y. E. Kou C. Li X. Tang B. H. Yao B. F. Hao G. X. . (2022). Developmental population pharmacokinetics-pharmacodynamics of meropenem in Chinese neonates and young infants: dosing recommendations for late-onset sepsis. Children 9:1998. doi: 10.3390/children9121998, 36553441 PMC9777159

[ref48] Yonwises W. Wacharachaisurapol N. Anugulruengkitt S. Maimongkol P. Treyaprasert W. (2021). Population pharmacokinetics of meropenem in critically ill infant patients. Int. J. Infect. Dis. 111, 58–64. doi: 10.1016/j.ijid.2021.08.031, 34419581

[ref49] Zylbersztajn B. Parker S. Navea D. Izquierdo G. Ortiz P. Torres J. P. . (2021). Population pharmacokinetics of vancomycin and meropenem in Pediatric extracorporeal membrane oxygenation support. Front. Pharmacol. 12:709332. doi: 10.3389/fphar.2021.709332, 34483917 PMC8411703

