# Supplementary material for: Population pharmacokinetic model for meropenem in pediatric patients: a systematic review
Source: Front Microbiol. 2026 Sep 17;17:1897237. doi: 10.3389/fmicb.2026.1897237 (PMC13627380; doi:10.3389/fmicb.2026.1897237)
Supplement: Supplementary file 1 [file Data_Sheet_1.pdf]

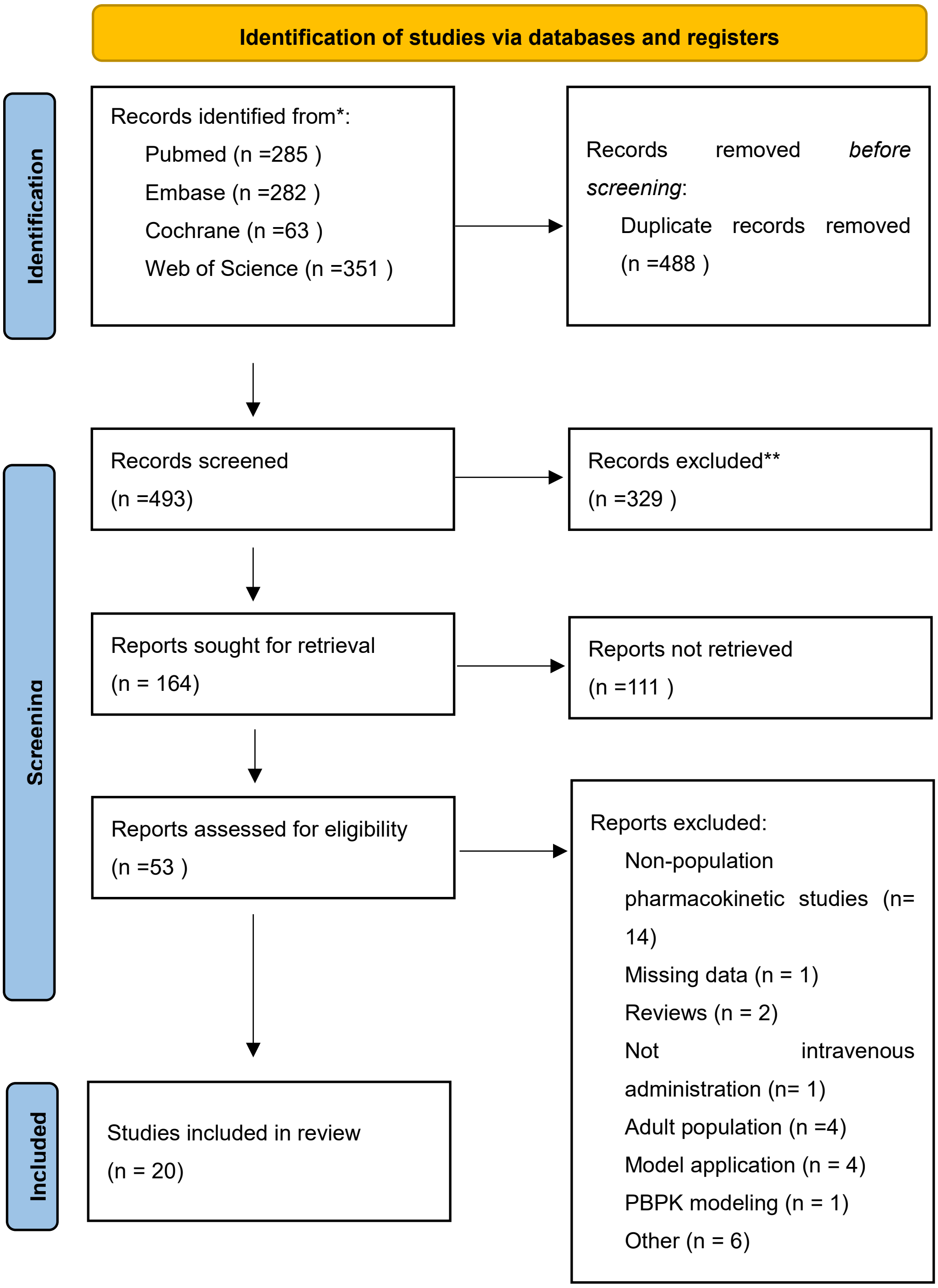

Figure S1 Study search and selection process

Source: Page MJ, et al. BMJ 2021;372:n71. doi: 10.1136/bmj.n71.

This work is licensed under CC BY 4.0. To view a copy of this license, visit https://creativecommons.org/licenses/by/4.0/

Table S1 Literature search strategy

**MEDLINE(PubMed)**	("Meropenem"[Mesh] OR "Meropenem"[tiab] OR "Merrem"[tiab] OR "SM 7338"[tiab] OR "SM-7338"[tiab] OR "SM7338"[tiab] OR "Ronem"[tiab] OR "Penem"[tiab] OR "3-(5-Dimethylcarbamoylpyrrolidin-3-ylthio)-6-(1-hydroxyethyl)-4-methyl-7-oxo-1-azabicyclo(3.2.0)hept-2-ene-2-carboxylic acid"[tiab]) AND ("Pharmacokinetics"[Mesh] OR "pharmacokinetic*"[tiab] OR "drug kinetic*"[tiab] OR "kinetic*"[tiab] OR "PK"[tiab] OR "pharmacodynamic*"[tiab] OR "PD"[tiab] OR "PK/PD"[tiab] OR "drug dynamic*"[tiab] OR "dynamic*"[tiab] OR "therapeutic drug monitor*"[tiab] OR "TDM"[tiab] OR "popPK"[tiab] OR "drug monitoring*"[tiab]) AND (("Child"[Mesh] OR "Infant"[Mesh] OR "Adolescent"[Mesh] OR "Infant, Newborn"[Mesh] OR "Pediatrics"[Mesh]) OR (pediatric*[tiab] OR paediatric*[tiab] OR child*[tiab] OR infant*[tiab] OR neonate*[tiab] OR newborn*[tiab] OR baby[tiab] OR babies[tiab] OR toddler*[tiab] OR adolescent*[tiab] OR teenage*[tiab] OR "young patient"[tiab]))
**Embase**	('meropenem'/exp OR 'meropenem':ti,ab OR 'merrem':ti,ab OR 'sm 7338':ti,ab OR 'sm-7338':ti,ab OR 'sm7338':ti,ab OR 'ronem':ti,ab OR 'penem':ti,ab OR '3-(5-dimethylcarbamoylpyrrolidin-3-ylthio)-6-(1-hydroxyethyl)-4-methyl-7-oxo-1-azabicyclo(3.2.0)hept-2-ene-2-carboxylic acid':ti,ab OR 'meropenem':ti,ab OR 'merrem':ti,ab OR 'sm 7338':ti,ab OR 'sm-7338':ti,ab OR 'sm7338':ti,ab OR 'ronem':ti,ab OR 'penem':ti,ab OR '3-(5-dimethylcarbamoylpyrrolidin-3-ylthio)-6-(1-hydroxyethyl)-4-methyl-7-oxo-1-azabicyclo(3.2.0)hept-2-ene-2-carboxylic acid':ti,ab) AND ('pharmacokinetics'/exp OR pharmacokinetic*:ti,ab OR 'drug kinetic*':ti,ab OR kinetic*:ti,ab OR pk:ti,ab OR pharmacodynamic*:ti,ab OR pd:ti,ab OR 'pk/pd':ti,ab OR 'drug dynamic*':ti,ab OR dynamic*:ti,ab OR 'therapeutic drug monitor*':ti,ab OR tdm:ti,ab OR poppk:ti,ab OR 'drug monitoring*':ti,ab) AND (('child'/exp OR 'infant'/exp OR 'newborn'/exp OR 'adolescent'/exp OR 'pediatrics'/exp) OR (pediatric*:ti,ab OR paediatric*:ti,ab OR child*:ti,ab OR infant*:ti,ab OR neonate*:ti,ab OR newborn*:ti,ab OR baby:ti,ab OR babies:ti,ab OR toddler*:ti,ab OR adolescent*:ti,ab))
**Web Of Science**	(ALL=Meropenem OR (TI=Meropenem OR AB=Meropenem) OR (TI=Merrem OR AB=Merrem) OR (TI="SM 7338" OR AB="SM 7338") OR (TI=SM-7338 OR AB=SM-7338) OR (TI=SM7338 OR AB=SM7338) OR (TI=Ronem OR AB=Ronem) OR (TI=Penem OR AB=Penem) OR (TI="3-(5-Dimethylcarbamoylpyrrolidin-3-ylthio)-6-(1-hydroxyethyl)-4-methyl-7-oxo-1-azabicyclo(3.2.0)hept-2-ene-2-carboxylic acid" OR AB="3-(5-Dimethylcarbamoylpyrrolidin-3-ylthio)-6-(1-hydroxyethyl)-4-methyl-7-oxo-1-azabicyclo(3.2.0)hept-2-ene-2-carboxylic acid")) AND (ALL=Pharmacokinetics OR (TI=pharmacokinetic* OR AB=pharmacokinetic*) OR (TI="drug kinetic*" OR AB="drug kinetic*") OR (TI=kinetic* OR AB=kinetic*) OR (TI=PK OR AB=PK) OR (TI=pharmacodynamic* OR AB=pharmacodynamic*) OR (TI=PD OR AB=PD) OR (TI="PK/PD" OR AB="PK/PD") OR (TI="drug dynamic*" OR AB="drug dynamic*") OR (TI=dynamic* OR AB=dynamic*) OR (TI="therapeutic drug monitor*" OR AB="therapeutic drug monitor*") OR (TI=TDM OR AB=TDM) OR (TI=popPK OR AB=popPK) OR (TI="drug monitoring*" OR AB="drug monitoring*")) AND (ALL=(pediatric* OR paediatric* OR child* OR infant* OR neonate* OR newborn* OR baby OR babies OR toddler* OR adolescent* OR teenage*))
**Cochrane Central Register of Controlled**	((MeSH descriptor: [Meropenem] explode all trees) OR (Meropenem OR Merrem OR "SM 7338"

**Trials**	OR SM-7338 OR SM7338 OR Ronem OR Penem OR "3-(5-Dimethylcarbamoylpyrrolidin-3-ylthio)-6-(1-hydroxyethyl)-4-methyl-7-oxo-1-azabicyclo(3.2.0)hept-2-ene-2-carboxylic acid"):ti,ab,kw)) AND ((pharmacokinetics OR pharmacokinetic* OR "drug kinetic*" OR kinetic*:ti,ab OR pk:ti,ab OR pharmacodynamic* OR pd OR pk/pd OR "drug dynamic*" OR dynamic* OR "therapeutic drug monitor*" OR tdm OR poppk OR "drug monitoring*"):ti,ab,kw) AND<br>(([mh Child] OR [mh Infant] OR [mh Adolescent] OR [mh Pediatrics]) OR (pediatric*:ti,ab,kw OR paediatric*:ti,ab,kw OR child*:ti,ab,kw OR infant*:ti,ab,kw OR neonate*:ti,ab,kw OR newborn*:ti,ab,kw OR baby:ti,ab,kw OR babies:ti,ab,kw OR toddler*:ti,ab,kw OR adolescent*:ti,ab,kw))

Table S2 Meropenem dosing and sampling information of the included studies

ID	Author	Dosage	Sampling time point
1	Du X, et al.	10, 20, or 40 mg/kg of meropenem administered intravenously over 5 or 30 minutes, dose intervals of 8, 12 or 24 hours were applied	Over an 8-hour period after the first dose
2	Bradley JS, et al.	Single doses of 10 mg/kg or 20 mg/kg administered over 30 minutes	Group 1: 1-2, 4-6, 10-12 hours postdose;<br>Group 2: 2-4, 6-8, 16-24 hours postdose
3	van den Anker JN, et al.	Single doses of 10, 20, or 40 mg/kg administered (30-minute intravenous infusion)	Pre-dose and at 0.25, 0.5, 0.75, 1, 2, 4, 8, 12 and 24 h post-infusion start
4	Ikawa K, et al.	9-44 mg/kg via 0.5- or 1-hour infusion	NR
5	Ohata Y, et al.	10 mg/kg q8 h, 20 mg/kg q8 h, 40 mg/kg q8 h by 0.5 h or longer infusion duration	At 15-25 min after the start of infusion or at 15 min to 6 h after end of infusion
6	Smith PB, et al.	Group 1 (GA<32 weeks, PNA<14 days):<br>20 mg/kg every 12 hours;<br>Group 2 (GA<32 weeks, PNA≥14 days) and Group 3 (GA≥32 weeks, PNA<14 days):<br>20 mg/kg every 8 hours;<br>Group 4 (GA≥32 weeks, PNA≥2 weeks):<br>30 mg/kg every 8 hours	The first group included infants with birthdays on an odd date (e.g., 1st, 3rd,5th) and who had blood (0.1 mL) collected any time in the 24 hours prior to the first dose and 0.5–1, 3–4, and 7–8 (Q8 hour dosing) or 10–12 (Q12 hour dosing) hours after completion of the first dose;<br>The second group included infants with birthdays on an even date (e.g., 2nd, 4th, 6th) and who were sampled any time in the 24 hours prior to the first dose and 1–2, 4–6, and 7–8 (Q8 hour dosing) or 10–12 (Q12 hour dosing) hours after completion of the first dose;<br>PK sampling at steady state (after the fourth dose) was performed any time in the 3 hours prior to the dose and 0.25 – 2.5 and 4 – 12 hours after completion of the dose.

7	Kongthavonsakul K, et al.	20 mg/kg/dose q8 h push over 15-20 s	Pre-dose and at 0 (immediately post-infusion), 1, 3, 5 and 7 hours after the second meropenem dose
8	Pettit RS, et al.	40 mg/kg meropenem every 8 hours, administered as a 3-hour	3–3.5, 4–5, 5–6, 7–8 hours after the initiation of the infusion
9	Cies JJ, et al.	180-2820 mg; 19-200 mg/kg	For 30-minute infusions: typically within 20 minutes to 2 hours after end of infusion; For prolonged (i.e.,3–4 hour infusions) or 24-hour continuous infusions: at the end of infusion and again 1 hour from the end of the infusion
10	Ohata Y, et al.	10 mg/kg q8h, 20 mg/kg q8 h, 40 mg/kg q8 h, infusion durations: 0.5 h	During or after completion of meropenem infusion or until as late as 6.5 h after completion of infusion
11	Wang ZM, et al.	An intravenous infusion over 0.5 to 1 h at 40 mg/kg of body weight/dose or 10 to 20 mg/kg/dose (q8 h) in patients with bacterial meningitis or other severe infections	Opportunistic sampling
12	Saito J, et al.	14.7–97.8 mg/kg/dose	Scavenged samples (88.3%) and prospective samples (11.7%), for prospective samples, 0 h, 1h or 3 h, 4 h
13	Wang Y, et al.	20 mg/kg or 40 mg/kg administered intravenously q8 h. Infusion durations were either 1 hour or a two-step 3-hour infusion (half dose over 0.5 h, second half over 2.5 h)	After at least 5 doses of meropenem. For children ≤ 30 kg: 0 (pre-dose), 5, 45, 90, 180, 360, and 480 minutes after the end of infusion; For children > 30 kg: 0 (pre-dose), 5, 15, 30, 60, 90, 120, 240, 360, 480 minutes after the end of infusion
14	Zylbersztajn B, et al.	Meropenem: 20–40 mg/kg every 8 or 12 h, administered as a 3-hour infusion	1 and 3 h post-infusion and before the next dose
15	Yonwises W, et al.	20–40 mg/kg/dose every 8 h, administered as an intravenous infusion over 1 or 3 h	At steady-state after the second dose; at 1 h (for 1-h infusion only), 2 h, 3 h (for 3-h infusion only), 4 h, 6 h, and 8 h post-dose (total of 5 samples per patient)
16	Wu YE, et al.	20-160 mg per dose, 9.3-40.4 mg/kg/dose	An opportunistic sampling strategy

17	Pokorná P, et al.	ECMO off: 20-40 mg/kg 2-3times a day;<br>ECMO on: loading dose 20 mg/kg + continuous infusion (30-120 mg/kg/day based on age)	NR (from TDM)
18	Butragueño-Laiseca L, et al.	Patients received 40 mg meropenem/kg of body weight every 8 hours intravenously. CKRT patients: the dosing interval was increased to 12 hours from the fourth dose.	Blood and urine samples: before (T0) and at 2, 4, 6, and 8 hours after starting the infusion (T2, T4, T6, T8) of the fourth dose.<br>For patients on CKRT, prefilter, postfilter, and effluent samples were also collected simultaneously.
19	Morales Junior R, et al.	20-40 mg/kg/dose (max 2000 mg/dose) every 8 h with 30-min infusion	Opportunistic residual blood samples collected during the first 7 days of meropenem therapy
20	Le J, et al.	20 mg/kg/dose every 8 h over 30 minutes as standard-of-care (maximum of 2000 mg/dose)	Intensive sampling: on Days 1–3 for cases: pre-dose, 0.5-, 1-, 2-, 4-, 8-h post-infusion;<br>Controls sampled: on Day 1 with fewer time points

Table S3. Quality assessment of the included studies

Items	Study ID																			
	1	2	3	4	5	6	7	8	9	10	11	12	13	14	15	16	17	18	19	20
**Title/abstract**																				
Drug and patient population	✓	✓	✓	✓	✓	✓	✓	✓	✓	✓	✓	✓	✓	✓	✓	✓	✓	✓	✓	✓
Name of the drug(s) studied	✓	✓	✓	✓	✓	✓	✓	✓	✓	✓	✓	✓	✓	✓	✓	✓	✓	✓	✓	✓
Route of administration	✗	✗	✓	✓	✗	✗	✓	✓	✓	✗	✗	✗	✗	✗	✗	✗	✗	✗	✓	✗
Population studied	✓	✓	✓	✓	✓	✓	✓	✓	✓	✓	✓	✓	✓	✓	✓	✓	✓	✓	✓	✓
Primary objective	✓	✓	✓	✓	✓	✓	✓	✓	✓	✓	✓	✓	✓	✓	✓	✓	✓	✓	✓	✓
Major findings	✓	✓	✓	✓	✓	✓	✓	✓	✓	✓	✓	✓	✓	✓	✓	✓	✓	✓	✓	✓
**Background**																				
Pharmacokinetic data	✓	✓	✓	✓	✓	✓	✓	✓	✓	✓	✓	✓	✓	✓	✓	✓	✓	✓	✓	✓
Study rationale	✓	✓	✓	✓	✓	✓	✓	✓	✓	✓	✓	✓	✓	✓	✓	✓	✓	✓	✓	✓
Specific objectives/hypotheses	✓	✓	✓	✓	✓	✓	✓	✓	✓	✓	✓	✓	✓	✓	✓	✓	✓	✓	✓	✓
**Methods**																				
Eligibility criteria	✓	✓	✓	✓	✓	✓	✓	✓	✓	✓	✓	✓	✓	✓	✓	✓	✓	✓	✓	✓
Co-administration or food	✗	✗	✗	✗	✗	✗	✓	✗	✗	✗	✗	✗	✗	✗	✗	✗	✗	✗	✗	✗
Dosing	✓	✓	✓	✓	✓	✓	✓	✓	✓	✓	✓	✓	✓	✓	✓	✓	✓	✓	✓	✓
Formulation	✓	✓	✓	✓	✓	✓	✓	✓	✓	✓	✓	✓	✓	✓	✓	✓	✓	✓	✓	✓
Sampling schedule	✓	✓	✓	✗	✓	✓	✓	✓	✓	✓	✓	✓	✓	✓	✓	✓	✗	✓	✓	✓
Bioanalytical methods	✓	✓	✓	✓	✓	✓	✓	✓	✓	✓	✓	✓	✓	✓	✓	✓	✓	✓	✓	✓
Methods for handling missing data	✗	✗	✗	✗	✗	✗	✗	✗	✗	✗	✗	✗	✗	✗	✗	✗	✗	✗	✗	✗
Modeling software	✓	✓	✓	✓	✓	✓	✓	✓	✓	✓	✓	✓	✓	✓	✓	✓	✓	✓	✓	✓
Statistical methods and software	✓	✓	✓	✓	✓	✓	✓	✓	✓	✓	✓	✓	✓	✓	✓	✓	✓	✓	✓	✓

Candidate structural models	✓	✓	✓	✓	✓	✓	✓	✓	✓	✓	✓	✓	✓	✓	✓	✓	✓	✓	✓	✓
Residual error structure	✓	✓	✗	✓	✓	✓	✓	✓	✓	✓	✓	✓	✓	✓	✓	✓	✓	✓	✓	✓
Methods for base model determination	✓	✓	✓	✓	✓	✓	✓	✓	✓	✓	✓	✓	✓	✓	✓	✓	✓	✓	✓	✓
Methods for base model evaluation	✓	✓	✓	✓	✓	✓	✓	✓	✓	✓	✓	✓	✓	✓	✓	✓	✓	✓	✓	✓
Covariates analysis strategy	✓	✓	✓	✓	✓	✓	✓	✓	✓	✓	✓	✓	✓	✓	✓	✓	✓	✓	✓	✓
Methods for final model evaluation	✓	✗	✓	✓	✓	✓	✓	✓	✓	✓	✓	✓	✓	✓	✓	✓	✓	✓	✓	✓
Distribution of individual model parameters	✓	✗	✓	✓	✓	✓	✓	✓	✓	✓	✓	✓	✓	✓	✓	✓	✓	✓	✓	✓
Estimation method(s) used	✓	✓	✓	✓	✓	✓	✓	✓	✓	✓	✓	✓	✓	✓	✓	✓	✓	✓	✓	✓
**Results**																				
Population characteristic	✓	✓	✓	✓	✓	✓	✓	✓	✓	✓	✓	✓	✓	✓	✓	✓	✓	✓	✓	✓
No. of subjects and observations	✓	✓	✓	✓	✓	✓	✓	✓	✓	✓	✓	✓	✓	✓	✓	✓	✓	✓	✓	✓
Schematic of the final model	✗	✗	✗	✗	✗	✗	✗	✗	✗	✓	✗	✗	✓	✗	✗	✗	✗	✗	✗	✗
Table of the final model parameters	✓	✗	✓	✓	✓	✓	✓	✓	✗	✓	✓	✓	✓	✓	✓	✓	✓	✓	✓	✓
Final model evaluation plots	✓	✗	✓	✗	✓	✓	✓	✓	✓	✗	✗	✓	✓	✓	✓	✓	✓	✓	✓	✓
Summary of the model-building process and the derived final model	✓	✓	✓	✓	✓	✓	✓	✓	✓	✓	✓	✓	✓	✓	✓	✓	✓	✓	✓	✓
Plot of concentrations vs. Time	✓	✗	✓	✓	✓	✓	✓	✗	✗	✓	✓	✓	✗	✗	✓	✓	✓	✓	✓	✓
**Discussion/conclusion**																				
Study limitations	✗	✗	✓	✗	✗	✗	✓	✓	✓	✗	✓	✓	✓	✓	✓	✓	✗	✓	✓	✓
Study findings	✓	✓	✓	✓	✓	✓	✓	✓	✓	✓	✓	✓	✓	✓	✓	✓	✓	✓	✓	✓
Compliance rate (%)	85.7	71.4	88.6	82.9	85.7	85.7	94.3	88.6	85.7	85.7	85.7	88.6	88.6	85.7	88.6	88.6	82.9	88.6	91.4	88.6

The detailed study ID can refer to Table S2.

Table S4 Meropenem clearance and distribution volume stratified by age and condition

Age group	Condition	Study	Parameter
Newborns	Sepsis	Wu YE, et al.	CL (L/h)=$1.63\times(CW/2680)^{0.75}\times(PNA/12)^{0.209}\times(GA/36.5)^{2.14}$<br>V (L)=$0.503\times(CW/2680)$
Neonates and infants	ECMO and CRRT	Pokorná P, et al.	CL (L/h)=$1.09\times(WT/7.88)$<br>V_d (L)=$3.98\times(WT/7.88)\times 2.04^{CRRT\_on}$
Neonates	Unspecified	Bradley JS, et al.	CL (L/h/kg) =0.041+0.040/SCr+0.003×(PCA-35)<br>V_d (L/kg)=0.40
Neonates	Unspecified	Van den Anker JN, et al.	CL (L/h)=$0.156+0.00112\times CL_{cr}+0.0925\times WT$<br>$V_c$ (L)=0.969
Infants and children	Crtically ill	Wang ZM, et al.	CL (L/h)=$4.79\times(WT/11)^{0.365}\times(CLcr/155.6)^{0.317}$<br>$V_1$ (L)=$3.68\times(WT/11)^{0.435}$<br>$V_2$ (L)=1.67
Infants and children	Spesis/spetic shock	Le J, et al.	CL (L/h)=$9.72\times(WT/70)^{0.75}\times([eGFR/140]^{0.169}$<br>$V_1$ (L)=$9.74\times(WT/70)\times(1+0.449\times SCys/0.45)$<br>$V_2$ (L)=$4.53\times(WT/70)$
Infants	Intra-abdominal infection	Smith PB, et al.	CL (L/h/kg)=0.122×[(0.5/SCr)0.267]×[(PMA/32.7)1.46]<br>V (L/kg)=0.460×(ALB/2.3)-0.444
Infants	Critically ill	Yonwises W, et al.	CL (L/h)=1.33×exp(0.126×(WT-5.70))+0.0085×(CLcr -112.43)<br>V_d (L)=2.27×exp(0.139×(WT-5.70))
Children	Severe infection	Kongthavonsakul K, et al.	V_c (L)=1.93<br>V_p (L)=2.13<br>CL (L/h)=6.53
Children	Cystic fibrosis	Pettit RS, et al.	CL (L/h/kg)=0.41×WT<br>V_c (L/kg)=12.29×WT/40.9
Children	Critically ill	Cies JJ, et al.	CL (L/h/kg)=0.419

			V_c (L/kg)=0.57
Children	Meningitis	Ohata Y, et al.	CL (L/h)=0.428×WT<br>V_c (L)=0.287×WT<br>V_p (L)=0.0537×WT
Children	ECMO and CRRT	Wang Y, et al.	CL (L/h)=7.6×(WT/12)$^{0.75}$×(1+(eCLcr-150)×0.0035)+0.257×Q×IF CRRT)<br>V_1 (L)=21.4×(WT/12)<br>V_2 (L)=0.025
Full age	Meningitis	Du X, et al.	CL(L/h) =4.22× (CLcr/53.35)$^{0.29}$× (WT/13.5)$^{0.86}$<br>V_c(L) =2.62× (WT/13.5)$^{1.06}$<br>V_p (L) =2.50× (WT/13.5)$^{0.30}$
Full age	Unspecified	Ikawa K, et al.	Cl_r (L/h)=0.254×WT<br>Cl_{nr} (L/h)=3.45<br>V_c (L)=0.272×WT<br>V_p (L)=0.228×WT
Full age	Unspecified	Ohata Y, et al.	CL (L/h)=0.526×WT<br>V_c (L)=0.292×WT<br>V_p (L)=0.108 × WT
Full age	Critically ill	Saito J, et al.	CL (L/h)=0.46×(eGFR/38.2)$^{0.19}$×(1+0.35)^{SIRS}×WT<br>V_{ci} (L)=0.49×(1+0.66)^{CRRT}×WT<br>V_p (L)=0.34×WT
Full age	ECMO and CRRT	Zylbersztajn B, et al.	CL (L/h/kg)=0.139<br>V_c (L/kg)=0.289
Full age	Critically ill with CRKT	Butragueño-Laiseca L, et al.	CL_r (L/h)=5.5 × (eGFR/95) × [AGE/(AGE+15.6)] (eGFR/95) × [AGE/(AGE+15.6)]<br>CL_{nr} (L/h)=0.91

Full age	Critically ill	Morales Junior R, et al.	CL_{CKRT} (L/h)=1.26 × θ_{FILT}, [θ_{FILT_MED}=1 (reference), θ_{FILT_High}=1.5, θ_{FILT_Low}=0.27]<br>V_1 (L)=4.75×WT/10<br>V_2 (L)=10.7<br>CL (L/h/70kg$^{0.75}$)=13.22×(WT/70)$^{0.75}$×(eGFR/140)$^{0.45}$×$e^{(\eta Cl+\eta IOV)}$× MF;<br>(MF=PMA^{Hill}/(PMA^{Hill}+PMA50^{Hill})])<br>V_1 (L/70kg)=16.45×(WT/70)×$e^{0.033\times Cum\%FB}$×$e^{\eta V1+\eta IOV}$,<br>V_2 (L/70kg)=7.66×(WT/70)

Note: Only drug clearance (CL) and distribution volume (V) captured in this table, other parameter and abbreviations can be referred to Table 4 in main text.
